# Highly efficient, selective, and stable photocatalytic methane coupling to ethane enabled by lattice oxygen looping

**DOI:** 10.1126/sciadv.ado4390

**Published:** 2024-06-28

**Authors:** Guangyao Zhai, Lejuan Cai, Jun Ma, Yihong Chen, Zehua Liu, Shenghe Si, Delong Duan, Shuaikang Sang, Jiawei Li, Xinyu Wang, Ying-Ao Liu, Bing Qian, Chengyuan Liu, Yang Pan, Ning Zhang, Dong Liu, Ran Long, Yujie Xiong

**Affiliations:** ^1^ Key Laboratory of Precision and Intelligent Chemistry, School of Chemistry and Materials Science, Department of Environmental Science and Engineering, National Synchrotron Radiation Laboratory, School of Nuclear Science and Technology, University of Science and Technology of China, Hefei, Anhui 230026, China.; ^2^ Sustainable Energy and Environmental Materials Innovation Center, Nano Science and Technology Institute, Suzhou Institute for Advanced Research, University of Science and Technology of China, Suzhou, Jiangsu 215123, China.; ^3^ Songshan Lake Materials Laboratory, Dongguan, Guangdong 523808, China.; ^4^ Anhui Engineering Research Center of Carbon Neutrality, School of Chemistry and Materials Science, Anhui Normal University, Wuhu, Anhui 241000, China.

## Abstract

Light-driven oxidative coupling of methane (OCM) for multi-carbon (C_2+_) product evolution is a promising approach toward the sustainable production of value-added chemicals, yet remains challenging due to its low intrinsic activity. Here, we demonstrate the integration of bismuth oxide (BiO_x_) and gold (Au) on titanium dioxide (TiO_2_) substrate to achieve a high conversion rate, product selectivity, and catalytic durability toward photocatalytic OCM through rational catalytic site engineering. Mechanistic investigations reveal that the lattice oxygen in BiO_x_ is effectively activated as the localized oxidant to promote methane dissociation, while Au governs the methyl transfer to avoid undesirable overoxidation and promote carbon─carbon coupling. The optimal Au/BiO_x_-TiO_2_ hybrid delivers a conversion rate of 20.8 millimoles per gram per hour with C_2+_ product selectivity high to 97% in the flow reactor. More specifically, the veritable participation of lattice oxygen during OCM is chemically looped by introduced dioxygen via the Mars-van Krevelen mechanism, endowing superior catalyst stability.

## Teaser

Precisely designed catalysts are used in an effective strategy for sustainable light-driven methane coupling.

## INTRODUCTION

Methane (CH_4_) is a promising feedstock for producing high-value-added chemical commodities in modern industries, which is the predominant constituent of natural gas, shale gas, and combustible ice (*1*). However, the conversion of CH_4_ is thermodynamically unfavorable due to its inert C─H bond (435 kJ mol^−1^) and high *T*_d_ symmetry, which means that activating CH_4_ molecules generally requires a high energy input. In addition, once CH_4_ is chemically activated, the corresponding intermediates (e.g., methyl species) become highly active, rendering the product distribution uncontrollable (*2*). Therefore, traditional oxidative CH_4_ conversion over thermocatalysis often confronts several adverse but inevitable predicaments including harsh reaction conditions, product overoxidation, and catalyst deactivation (*3*).

To this end, light-driven CH_4_ conversion through a photocatalytic process, which requires only renewable solar energy as an energy source, offers an alternative approach for converting CH_4_ into value-added products under mild conditions (*4*). Although the nonoxidative coupling of methane has received ongoing attention for generating stoichiometric ethane (C_2_H_6_) and hydrogen, the reaction efficiency is limited by the high free energy barrier (68.6 kJ mol^−1^) (*5*, *6*). Alternatively, oxidative coupling of methane (OCM) in the presence of O_2_ is a promising approach for achieving high CH_4_ conversion activity as it can bypass thermodynamic limitations (*7*, *8*). In the typical photocatalytic OCM process, the photogenerated holes contribute to the activation of CH_4_ molecules, while the photogenerated electrons turn to reduce O_2_ to superoxide radicals as oxidants (*9*). Nevertheless, such a process still confronts several technical drawbacks that hamper the overall efficiency of CH_4_ conversion to value-added C_2+_ products (*10*, *11*). The first fundamental factor for dominating the conversion efficiency is the activation of CH_4_ molecules (*12*–*14*). Catalytic site engineering is thus essential for maneuvering the interaction between CH_4_ and the reaction center. In comparison to the sole metal sites in conventional catalyst hosts, lattice oxygen-mediated dual sites (i.e., metal-oxygen active sites) can act as effective centers to actuate reactants and stabilize specific intermediates (*15*, *16*). Accordingly, two key points deserve sufficient attention. One key aspect of this process is that for lattice oxygen to participate in a chemical reaction, it must be substantially activated, which is determined by the electronic states of the catalyst host. The other is the steady lattice oxygen looping during the catalytic process, following the Mars-van Krevelen mechanism (*17*–*19*). In terms of OCM, such chemical looping is possibly accomplished by introducing O_2_ (*20*).

Another crucial parameter for improving CH_4_ conversion efficiency is the selectivity of the desired C_2+_ products. In the photocatalytic OCM process, once CH_4_ is activated, methyl species are proposed to form (*21*). Unfortunately, the chemically active nature of methyl species renders their overoxidation uncontrollable, especially in the existence of free superoxide radicals, reducing the selectivity of C_2+_ product (typically lower than 90%) (*10*, *22*–*26*). Under this scenario, subtle manipulation of the reaction pathway is highly imperative for accessing both CH_4_- and O_2_-involved intermediates to avoid unfavorable overoxidation. Intuitively, the formed methyl species should be transferred far from the oxidizing environment to facilitate C─C coupling, while the formation of free superoxide radicals should be avoided. In terms of methyl species, it is underlined that Au cocatalysts can efficiently facilitate its transfer at the catalyst surface. For instance, Ye and colleagues demonstrated an Au-ZnO/TiO_2_ hybrid photocatalyst to endow a high C_2_H_6_ selectivity of 90% (*9*). Considering the matter of CH_4_ activation, O_2_ is expected to participate in chemical lattice oxygen looping, whereas the active oxygen species (i.e., activated lattice oxygen) are thus localized at the catalyst surface rather than being free to attack the methyl species.

Leveraging the underlying understanding of the photocatalytic OCM process, we believe that a multifunctional photocatalyst host is required to achieve high CH_4_ conversion activity and product selectivity, whose different components contribute to CH_4_ activation and C─C coupling through rational catalytic site engineering, respectively. Here, taking TiO_2_ as a photocatalyst substrate, we report that the integration of BiO_x_ and Au can lead to excellent photocatalytic OCM performance. Owing to the elevated electronic band of BiO_x_, lattice oxygen is effectively activated to participate in the C─H dissociation of CH_4_ via the H_3_C*-Bi-O-*H configuration assisted by photogenerated holes. The adjacent Au guides the transfer of methyl species from the Bi site to avoid undesirable overoxidation and promotes the C─C coupling to generate C_2+_ products (dominant C_2_H_6_). The lattice oxygen in BiO_x_ virtually reacts to generate H_2_O, which is chemically looped by the introduced O_2_ at an adequate concentration through the Mars-van Krevelen mechanism, retaining good catalyst durability. In a flow reactor, a remarkable CH_4_ conversion rate of 20.8 mmol g^−1^ hour^−1^ is presented over the optimal Au/BiO_x_-TiO_2_ hybrid, together with a C_2+_ product selectivity as high as 97% and a specific C_2_H_6_ production rate of 9.6 mmol g^−1^ hour^−1^. Moreover, a turnover number (TON) up to 94,675 with respect to the Au site is also achieved during the continuous 50-hour durability test, demonstrating the state-of-the-art performance of photocatalytic OCM.

## RESULTS

**Fig. 1.**
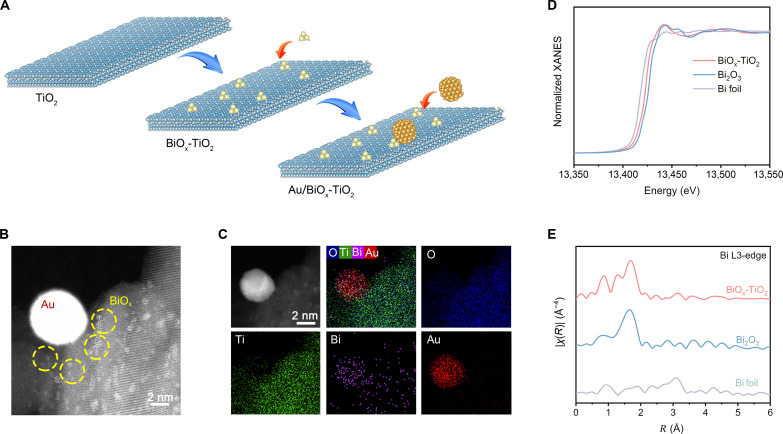
Structural characterizations. (**A**) Schematic illustration of synthetic process of Au/BiO_x_-TiO_2_. (**B**) High-angle annular dark-field scanning transmission electron microscope image of Au/BiO_x_-TiO_2_, where BiO_x_ clusters are denoted by yellow dotted circles. (**C**) Elemental energy-dispersive x-ray spectroscopy mapping of Au/BiO_x_-TiO_2_ with O (blue), Ti (green), Bi (purple), and Au (red). (**D**) Normalized Bi L_3_-edge x-ray absorption near-edge structure (XANES) spectra and (**E**) k^2^-weighted Fourier-transform Bi L_3_-edge x-ray absorption fine structure spectra for BiO_x_-TiO_2_ in reference to Bi_2_O_3_ and Bi foil.

The Au/BiO_x_-TiO_2_ catalyst was synthesized by a two-step photodeposition method, as illustrated in Fig. 1A. First, Bi^3+^ ions were electrostatically adsorbed on TiO_2_ nanosheets by virtue of the negative zeta potential (−6.7 mV) of the TiO_2_ surface (fig. S1). Then, the Bi^3+^ species were reduced to metallic Bi species under light irradiation in an Ar atmosphere, which is thermodynamically feasible based on the energy band structure of TiO_2_ (fig. S2) and redox potential of Bi^3+^/Bi^0^ (fig. S3). Afterward, the metallic Bi was easily oxidized to BiO_x_ after exposure to air (fig. S4). The zeta potential of the as-obtained BiO_x_-TiO_2_ sample turns out to be positive at 16.6 mV, thus supplying the electrostatic interaction to adsorb the AuCl_4_^−^ anion. Afterward, Au was photoreduced on a BiO_x_-TiO_2_ substrate, forming the Au/BiO_x_-TiO_2_ hybrid. The loading amounts of Bi and Au are tunable, as determined by inductively coupled plasma mass spectrometry (ICP-MS; see table S1). Structural characterizations suggest that the phase and morphology of the TiO_2_ substrate are not altered after the deposition of BiO_x_ and Au (figs. S5 to S8). To further confirm the structural states of BiO_x_ and Au, we conducted high-angle annular dark-field scanning transmission electron microscope (HAADF-STEM) measurements. The related images show the homogeneous dispersion of BiO_x_ nanoclusters (NCs) in both Au/BiO_x_-TiO_2_ and BiO_x_-TiO_2_ samples (Fig. 1B and fig. S7). In addition, Au nanoparticles (NPs) with the size of 4 to 8 nm are surrounded by BiO_x_ NCs in Au/BiO_x_-TiO_2_ (Fig. 1B and fig. S8). The absence of metallic Au peak in the x-ray diffraction (XRD) pattern may be ascribed to the low loading amount of only 0.2 wt %. Elemental analysis through energy-dispersive x-ray spectroscopy (EDS) mapping further illustrates the aforementioned dispersion of BiO_x_ NCs and Au NPs on the TiO_2_ substrate (Fig. 1C), implying the tight combination between BiO_x_ and Au. Ultraviolet-visible (UV-vis) diffuse reflectance spectra show that the absorption edge of BiO_x_-TiO_2_ slightly trails compared with that of bare TiO_2_ (fig. S9), attributed to the BiO_x_ NCs deposited on TiO_2_. The absorption peak at approximately 540 nm also clearly confirms the presence of Au NPs, which is derived from the surface plasmon resonance character.

X-ray photoelectron spectroscopy (XPS) was subsequently used to confirm the valence states and electronic interaction. In comparison to those of bare TiO_2_, the high-resolution Ti 2p peaks of both BiO_x_-TiO_2_ and Au/BiO_x_-TiO_2_ shift toward lower binding energies (BEs) (fig. S10), suggesting the electron shift from BiO_x_ to the TiO_2_ substrate. In the meantime, Bi 4f XPS spectra dominantly show the characteristic peaks of oxidized Bi^3+^ species in BiO_x_ NCs (fig. S11), together with secondary metallic Bi signals (*27*, *28*). To be specific, the Bi 4f peaks blueshift toward higher BEs after Au deposition, indicative of further electron transfer from BiO_x_ to Au. Such electronic interactions are also consolidated by the reverse redshift in the Au 4f XPS spectra (fig. S12) (*29*). From the catalytic point of view, the electron-deficient state of Bi species can facilitate the chemisorption and activation of CH_4_ molecules for oxidative conversion. The atomic structure of BiO_x_ NCs is further untangled by synchrotron radiation-based x-ray absorption fine structure (XAFS) spectroscopy (*30*). We measured BiO_x_-TiO_2_ rather than Au/BiO_x_-TiO_2_ because of the overlap between Bi and Au absorption edge, which does not influence our discussion. X-ray absorption near-edge structure spectroscopy (Fig. 1D) shows that the Bi L_3_-edge of BiO_x_ is located between Bi foil and Bi_2_O_3_ references, implying that the valence of the Bi species is between 0 and +3. This is reasonable due to its NC nature. To affirm this argument, the extended XAFS (EXAFS) spectra were Fourier-transformed (Fig. 1E), revealing the major peak (1.69 Å) for the Bi─O scattering path of BiO_x_. The related fitting results (fig. S13 and table S2) show that the coordination number (CN) of the Bi─O bond in BiO_x_ is 2.2, which is lower than that of the reference Bi_2_O_3_ (CN = 3). A lower CN is indicative of a coordinately unsaturated feature with abundant oxygen vacancies (OVs) in BiO_x_ NCs. Such OV-rich character is further strengthened by electron paramagnetic resonance (EPR), whereas both BiO_x_-TiO_2_ and Au/BiO_x_-TiO_2_ exhibit the enhanced characteristic peak at *g* = 2.001 with respect to bare TiO_2_ (fig. S14).

**Fig. 2.**
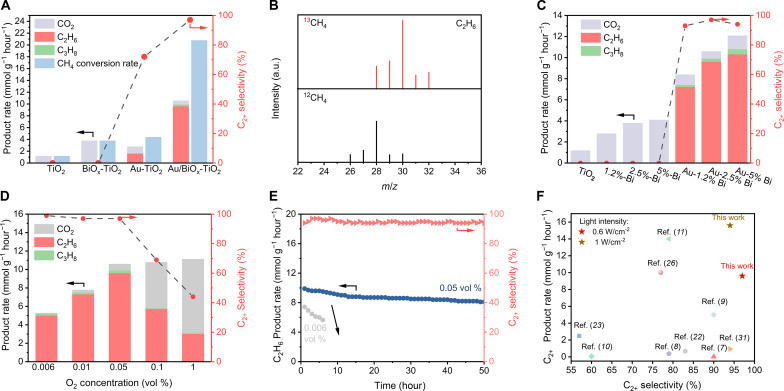
Activity evaluations for photocatalytic OCM. (**A**) The generation rate and selectivity of CH_4_ oxidative products over various catalysts. (**B**) Mass spectra of C_2_H_6_ produced over Au/BiO_x_-TiO_2_ using ^12^CH_4_ and ^13^CH_4_ as reactants, respectively. a.u., arbitrary units. (**C**) Photocatalytic activity and selectivity of C_2+_ products over BiO_x_-TiO_2_ and Au/BiO_x_-TiO_2_ catalysts with different Bi loading amounts. (**D**) The generation rates of various products over Au/BiO_x_-TiO_2_ under different O_2_ concentrations. (**E**) Stability assessment of Au/BiO_x_-TiO_2_ in the flow reactor. (**F**) Performance comparison with the recently representative works [i.e., Ag-HPW/TiO_2_ (*7*), Ag-AgBr/TiO_2_ (*8*), Au-ZnO/TiO_2_ (*9*), Cu_0.1_Pt_0.5_/PC-50 (*10*), Au_1_Ag-ZnO (*11*), Au/ZnO (*22*), PdCu/TiO_2_ (*23*), Au_0.05_-Pd_0.05_/TiO_2_ (*26*), and Pd_1_/TiO_2_ (*37*)] for photocatalytic oxidative methane conversion at normal temperatures.

Upon resolving the structure of the as-obtained catalyst, we evaluated the activity of the photocatalytic OCM in a continuous flow reactor (fig. S15). As shown in Fig. 2A, the introduction of BiO_x_ into TiO_2_ (i.e., BiO_x_-TiO_2_ catalyst) apparently promotes the photocatalytic activity, increasing the CH_4_ conversion rate (3.8 mmol g^−1^ hour^−1^) greater than three times higher than that of bare TiO_2_ (1.2 mmol g^−1^ hour^−1^). This finding implies that BiO_x_ NCs can effectively activate CH_4_ molecules. Unfortunately, valueless CO_2_ is detected as a by-product, meaning uncontrollable overoxidation. In sharp contrast, C_2+_ products (dominant C_2_H_6_) were detected over the Au-TiO_2_ catalyst with a selectivity of 72%, implying that Au plays an indispensable role in the C─C coupling process. However, the CH_4_ conversion rate over Au-TiO_2_ is still unsatisfactory (4.4 mmol g^−1^ hour^−1^). When we integrated BiO_x_ NCs and Au NPs into the TiO_2_ host, the related catalyst (i.e., Au/BiO_x_-TiO_2_ hybrid) well-emerged the synergistic effect, contributing to an improved CH_4_ conversion rate of up to 20.8 mmol g^−1^ hour^−1^ as well as a C_2+_ product selectivity high to 97%. Given that C_2_H_6_ is the main product, its production rate was specifically determined to be 9.6 mmol g^−1^ hour^−1^. In these regards, we surmise that BiO_x_ NCs effectively activate CH_4_ molecules to facilitate C─H bond dissociation, while the interacting Au NPs benefit the subsequent C─C coupling to generate C_2+_ products and limit unfavorable overoxidation, which will be comprehensively discussed in the following sections. In addition, we further performed the photocatalytic tests using various reference samples including Bi_2_O_3_, Au/Bi_2_O_3_, and Au/BiO_x_ loaded on insert supports (SiO_2_ and ZrO_2_), all of which cannot trigger the OCM reaction. Such contrast experiments suggest that the BiO_x_ NCs accept the electrons from the photocatalyst host rather than generate electrons by themselves. Specifically, H_2_O rather than H_2_ is detected as the product of CH_4_ conversion, indicating that the reaction is an OCM process involving the participation of oxygen. Accordingly, we determined the oxygen balance to be about 98% (see details in note S1), suggesting the stoichiometric OCM reaction. Controlled experiments were performed to corroborate the necessity of a light-driven catalytic OCM process. In the dark condition, OCM cannot be triggered even at an elevated reaction temperature of 423 K (fig. S16), illustrating the light-driven rather than thermal-driven nature of OCM. The photocatalytic activity is directly proportional to the light intensity, whose C_2_H_6_ production rate can reach 15.6 mmol g^−1^ hour^−1^ at a strong light intensity of 1 W cm^−2^ with a retained selectivity of 95% (fig. S17). The wavelength-dependent apparent quantum efficiency (AQE) for OCM was also studied. The Au/BiO_x_-TiO_2_ catalyst achieved an AQE of 3.8% under 365-nm monochromatic light illumination (fig. S18). In addition, the generation rate of C_2+_ products over Au/BiO_x_-TiO_2_ can still be maintained with the amplification of catalyst usage under appropriate reaction conditions (fig. S19). To confirm the origin of the product, isotope-labeled ^13^CH_4_ was used as the feeding source to conduct the photocatalytic experiments. The observed product was checked by mass spectrometry (MS), whose dominant signal at mass/charge ratio (*m/z*) = 30 clearly suggested that the ^13^C_2_H_6_ products were generated from the light-driven OCM process (Fig. 2B).

Given the acquired reaction process of light-driven OCM over Au/BiO_x_-TiO_2_, the photocatalysts were then optimized correspondingly. The effect of BiO_x_ NCs on the performance was studied by changing the loading amount. As shown in Fig. 2C, the CH_4_ conversion gradually improves along with increasing BiO_x_ loading for both BiO_x_-TiO_2_ and Au/BiO_x_-TiO_2_, indicating that CH_4_ activation is closely related to BiO_x_ NCs. However, the activity is close to the maximum with Bi loading of 2.5 wt %, which can be interpreted by the incomplete oxidation of Bi species (see the recorded metallic Bi peak in the XRD pattern in fig. S20). Therefore, the oxidized BiO_x_ NCs are regarded as the genuine CH_4_ activation site. In addition, the deposited Au amount was also investigated by fixing the optimized Bi amount at 2.5 wt % (fig. S21), whereas 0.2 wt % is considered the optimal Au loading amount from the viewpoint of catalytic performance and atomic utilization efficiency.

Specifically, the feeding O_2_ concentration during photocatalytic experiments is vital for both the CH_4_ conversion rate and the product selectivity. As displayed in Fig. 2D, increasing the O_2_ concentration positively affects the catalytic performance at a low concentration of 0.05 vol %, at which point the selectivity of C_2+_ products is well retained. However, a further increment in O_2_ concentration results in marked selectivity bleaching induced by severe overoxidation to CO_2_. For instance, the C_2+_ product selectivity rapidly decreases from 97 to 44% when O_2_ concentration is increased from 0.05 to 1 vol %. The O_2_ concentration–dependent performance, especially for selectivity, may be attributed to the distinct oxygen-active species that oxidize CH_4_ molecules via different reaction pathways. The optimized O_2_ feeding concentration was determined to be 0.05 vol %, achieving a C_2+_ product selectivity of 97% and a specific C_2_H_6_ production rate of 9.6 mmol g^−1^ hour^−1^. The catalyst durability is also highly related to the feeding O_2_ concentration. As shown in Fig. 2E, the catalytic performance of Au/BiO_x_-TiO_2_ was well maintained after the continuous 50-hour test at an O_2_ concentration of 0.05 vol %. In comparison, the durability is very poor when the O_2_ concentration lowers to 0.006 vol %, whereas the C_2_H_6_ production rate markedly declines by about 40% within 6 hours. Hence, a certain concentration of O_2_ is regarded to dominantly govern the catalyst stability. As experimentally confirmed, the XRD and transmission electron microscopy (TEM) results of the Au/BiO_x_-TiO_2_ post-catalyst indicate the unchanged phase and morphology after the durability test at 0.05 vol % O_2_ concentration (figs. S22 and S23). XPS spectra also show that the Bi species have a dominant valence of +3 (fig. S24), meaning the electronic states are also retained. After the 50-hour durability test, the overall TON values based on Bi and Au sites were calculated to be as high as 17,897 and 94,675, respectively. By virtue of the high activity, selectivity, stability, and atomic economy, our optimal Au/BiO_x_-TiO_x_ catalyst exhibits competitive performance in terms of OCM with respect to other recently reported catalyst alternatives, especially for the state-of-the-art C_2+_ product selectivity, as underlined in Fig. 2F and tables S3 and S4.

**Fig. 3.**
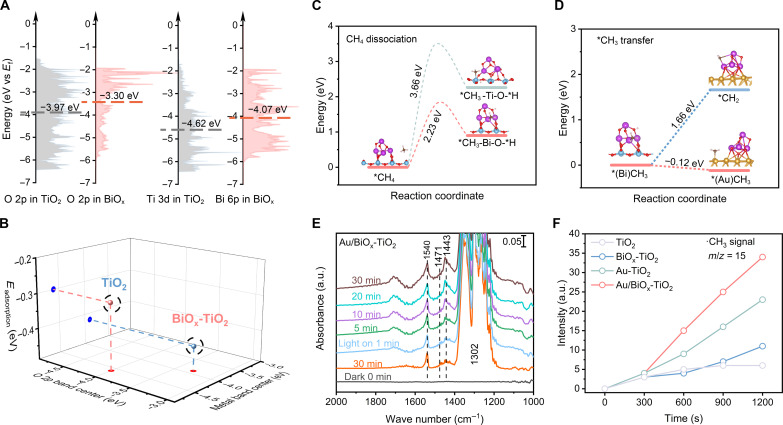
In-depth investigations of the reaction pathway. (**A**) The PDOS diagram of O 2p bands in TiO_2_ and BiO_x_-TiO_2_, Ti 3d and Bi 6p bands in BiO_x_-TiO_2_. The dotted lines are the determined band centers. (**B**) The CH_4_ adsorption energy over TiO_2_ and BiO_x_-TiO_2_ models connected with O 2p band center and metal band center. (**C**) The determined free energy diagrams for C─H dissociation of CH_4_ on Ti─O and Bi─O sites over the BiO_x_-TiO_2_ model, respectively. (**D**) The determined free energy diagrams for *CH_3_ transfer from Bi to Au site and further dissociation to *CH_2_ at Bi site, respectively. (**E**) In situ diffuse reflectance infrared Fourier transform spectroscopy spectra for photocatalytic OCM process with different reaction times over Au/BiO_x_-TiO_2_. (**F**) The determined signal intensity of ·CH_3_ radical (*m/z* = 15) over various catalysts through in situ synchrotron radiation photoionization MS measurements at a photon energy of 10.7 eV.

Intuitively, effective adsorption and activation of CH_4_ is the prerequisite to turning on the following OCM process. The CH_4_ temperature programmed desorption (CH_4_-TPD) experiments (fig. S25) showed that the deposited BiO_x_ NCs promoted CH_4_ adsorption, as both the physisorption and chemisorption peaks over BiO_x_-TiO_2_ and Au/BiO_x_-TiO_2_ were located at higher temperatures than those over bare TiO_2_. To fundamentally understand the profound influence of BiO_x_ NCs on molecular adsorption and activation, theoretical simulations were performed. The models of bare TiO_2_ and BiO_x_-TiO_2_ are shown in figs. S26 and 27, respectively, together with their CH_4_ adsorption configurations (figs. S28 and 29). Judging from the results, BiO_x_-TiO_2_ model gives a higher CH_4_ adsorption energy of −0.42 eV than that of the TiO_2_ model (−0.30 eV) (fig. S30), corroborating the promoted interaction between the adsorption site and CH_4_ molecule. Such promotion can be rationalized by the distinct electronic states of TiO_2_ and BiO_x_ adsorption host. Briefly, Ti─O bonds with Ti 3d and O 2p orbital hybridization are proposed for TiO_2_, while the 6p orbitals of Bi center contribute to interactions with oxygen ligands on BiO_x_. To decipher the electronic structure, the project density of states (PDOS) was calculated, as diagramed in Fig. 3A. The related band centers (ε) were determined, signifying that both Bi 6p and O_Bi_ 2p band in BiO_x_ upshift toward the Fermi level (*E*_F_) related to Ti 3d band and O_Ti_ 2p band in TiO_2_, respectively. The elevated electronic band in BiO_x_ thus benefits the molecular adsorption according to the well-established band center theory (*31*, *32*), as related in Fig. 3B. More specifically, the O 2p band distribution in BiO_x_ is dominantly located closer to *E*_F_ than that in TiO_2_. Taking the elevated ε_O 2p_ together (−3.30 versus −3.97 eV for BiO_x_ and TiO_2_), we can claim that the lattice oxygen ligands in BiO_x_ are more thermodynamically feasible to hole-doping for its activation, which plays a vital role in the following reaction. To affirm this argument, C─H bond dissociation (i.e., *CH_4_ → *CH_3_ + *H) was simulated at both Ti─O and Bi─O sites over BiO_x_-TiO_2_ model (Fig. 3C) (*33*). The dissociated *CH_3_ and *H species are determined to bond with Ti/Bi and O sites, respectively. The related Gibbs free energy (Δ*G*) was calculated to be only 0.90 eV for the Bi─O site. As compared, a much higher Δ*G* value of 2.26 eV is observed for the Ti─O site. Moreover, the kinetics of C─H dissociation were also investigated by determining the free energy barrier (Δ*G*^‡^) with the transition state. The Δ*G*^‡^ for C─H dissociation on the Ti─O site is determined to be 3.66 eV (fig. S31), apparently higher than that on the Bi─O site (Δ*G*^‡^ = 2.23 eV; fig. S32). Such free energy differences indicate that the C─H dissociation of CH_4_ is facilitated over BiO_x_ with respect to TiO_2_, which is the determinant for the activity of CH_4_ conversion. Fundamentally, the bond dissociation process is largely governed by the regulated chemical affinities of intermediates. The elevated electronic bands in BiO_x_ enable both Bi and O sites more chemically active, exhibiting great binding strength toward *CH_3_ and *H, respectively, thereby favoring the C─H dissociation. Afterward, the following process was also investigated. As Au was experimentally proven to decisively turn the product from CO_2_ to C_2_H_6_, its impact on *CH_3_ species was resolved theoretically by constructing a specific Au-BiO_x_ model (fig. S33). As illustrated in Fig. 3D, *CH_3_ species transfer spontaneously from the Bi site to the adjacent Au site with a negative Δ*G* of −0.12 eV. In comparison, further deprotonation to *CH_2_ species (simulating the further oxidation scene) on the Bi site is unfavorable with Δ*G* as high as 1.66 eV. Given that Au is the ideal site for C─C coupling (*4*, *9*, *22*), we can conclude that the presence of Au NPs in catalysts crucially prevents CH_4_ overoxidation and guides the C─C coupling process.

To further look into the reaction pathway, we experimentally performed in situ diffuse reflectance infrared Fourier transform spectroscopy (DRIFTS) measurements to monitor the intermediate evolution over Au/BiO_x_-TiO_2_ and BiO_x_-TiO_2_ catalysts under the light irradiation, as shown in Fig. 3E and fig. S34, respectively. The robust peak at 1302 cm^−1^ is ascribed to the C─H deformation vibration of the free CH_4_ molecule (*34*). After CH_4_ adsorption for 30 min under the dark condition, a characteristic peak at 1540 cm^−1^ appears, attributed to the C─H symmetric deformation vibrational mode of chemisorbed CH_4_ on the catalyst surface (*35*, *36*). Upon light irradiation, several characteristic signals emerge immediately, meaning that both Au/BiO_x_-TiO_2_ and BiO_x_-TiO_2_ can effectively trigger the light-driven CH_4_ conversion. The signals at 1471/1443 cm^−1^ are indexed to the deformation vibrational modes of CH_2_/CH_3_ species, indicating the CH_4_ dissociation (*15*, *37*). However, the following reaction intermediates undergo distinct evolution during light irradiation. Au/BiO_x_-TiO_2_ exhibited a gradually increasing signal at 1443 cm^−1^ (Fig. 3E), suggesting the aggregation of CH_3_ species (i.e., formation of C_2_H_6_). In comparison, such CH_3_ species are continuously consumed over BiO_x_-TiO_2_ (fig. S34). Meanwhile, the stretching vibrational modes associated with methoxy species (1084 cm^−1^), C─O bonds (1023 cm^−1^), and C═O bonds (1593 cm^−1^) are also recognizable for BiO_x_-TiO_2_ with considerable signal intensity (*15*, *37*–*39*), implying severe oxygenation of the CH_4_ molecule. These oxygen-involved signals hardly appear over Au/BiO_x_-TiO_2_, underlining the unfavorable oxygenation is largely alleviated to contribute to the high selectivity of C_2_H_6_ product.

To further resolve the molecular evolution, CH_3_ radical, a key but unstable intermediate during OCM, was dynamically monitored through in situ synchrotron radiation photoionization MS (SR-PIMS) over various catalysts (Fig. 3F and figs. S35 and 36) (*24*). When the photon energy was elevated to 10.7 eV, the CH_3_ signal at *m/z* = 15 was recognizable for all catalysts and increased along with the detection time. Specifically, the measured signal intensity is quite different over different catalysts. The intensity of BiO_x_-TiO_2_ is only slightly greater than that of bare TiO_2_ (Fig. 3F), indicating that overoxidation can hardly be avoided over the BiO_x_ site although it can promote the CH_4_ activation. Meanwhile, Au-TiO_2_ in turn delivers a much greater intensity than BiO_x_-TiO_2_, although they experimentally exhibit comparable CH_4_ conversion rates (i.e., 3.8 versus 4.4 mmol g^−1^ hour^−1^; see Fig. 2A). This means that ·CH_3_ radical can be well stabilized by Au, facilitating the C─C coupling process rather than the oxidative dehydrogenation. In this regard, the integrated Au/BiO_x_-TiO_2_ hybrid shows a much higher ·CH_3_ intensity, underlining that the improved evolution of ·CH_3_ species by the BiO_x_ site can be well retained by the adjacent sites. By combining the theoretical simulations with spectroscopic measurements, we can untangle the advance of Au/BiO_x_-TiO_2_ in the OCM process that BiO_x_ NCs promote effective CH_4_ activation and related C─H bond dissociation, while Au NPs benefit from the following C─C coupling to avoid overoxidation, thereby contributing to the remarkable overall CH_4_ conversion efficiency together with high C_2+_ product selectivity.

**Fig. 4.**
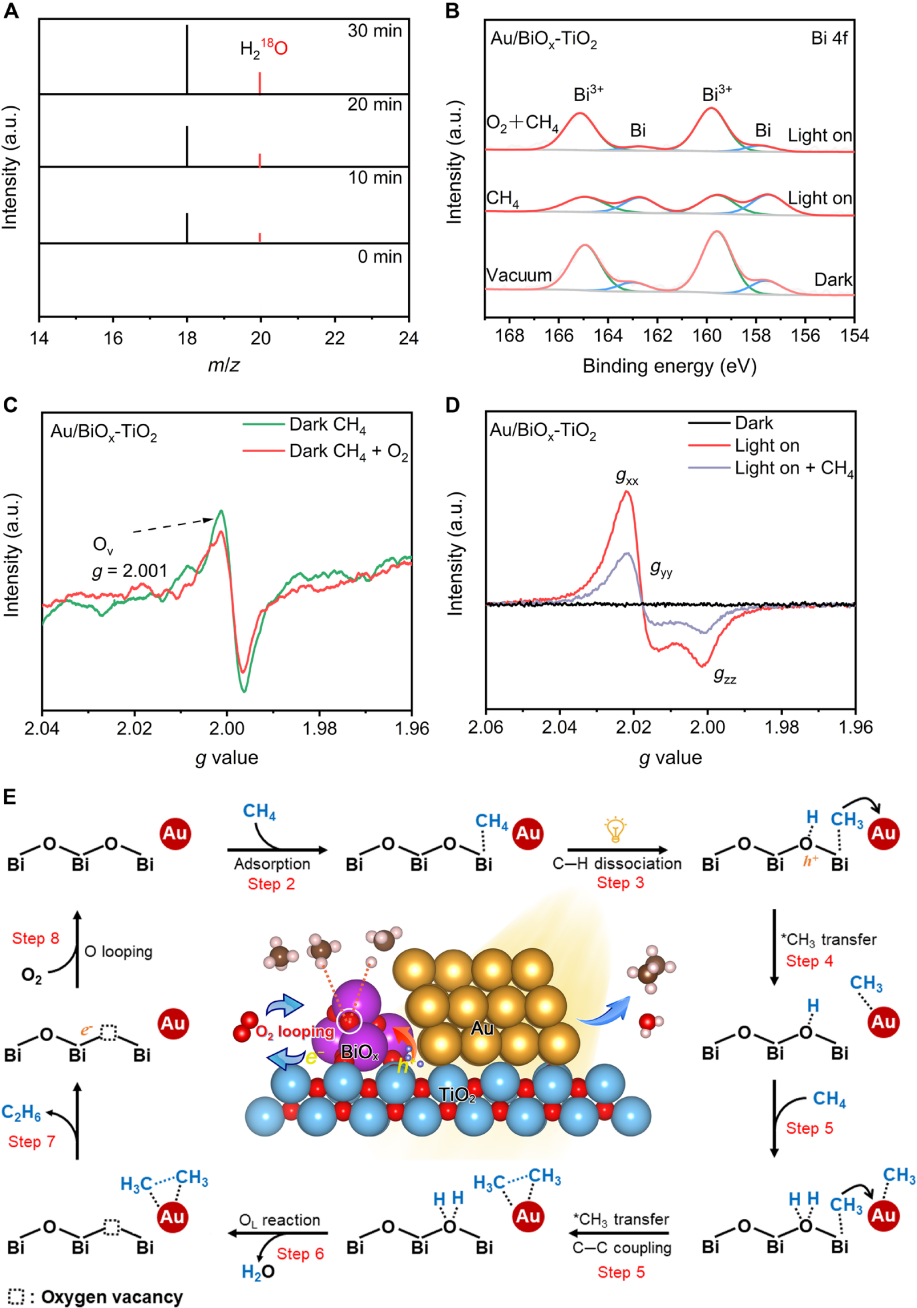
Understanding the lattice oxygen looping during the OCM process. (**A**) Photoionization mass spectra of H_2_O product using Au/Bi^18^O_x_-TiO_2_ nanocomposite at a photon energy of 12.8 eV. (**B**) In situ near ambient pressure XPS of Bi 4f spectra over Au/BiO_x_-TiO_2_ under 10-min light irradiation in different atmospheres. (**C**) Electron spin resonance (ESR) spectra of Au/BiO_x_-TiO_2_ under the dark condition in CH_4_ and CH_4_ + O_2_ atmosphere, respectively. (**D**) In situ ESR spectra of Au/BiO_x_-TiO_2_ under the dark condition at O_2_ atmosphere (black line) and under the light irradiation condition at O_2_ atmosphere (red line) and CH_4_ + O_2_ atmosphere (blue line), respectively. (**E**) Proposed reaction pathway for the light-driven OCM to C_2_H_6_ product over Au/BiO_x_-TiO_2_ at the proper O_2_ concentration.

According to the calculated PDOS diagrams, it is surmised that the lattice oxygen ligands in BiO_x_ NCs act as the vital knob on CH_4_ activation, thereby on the CH_4_ conversion activity. Given that H_2_O was also detected as the product, we designed ^18^O isotope labeling experiments to affirm this conjecture. Specifically, the photodeposition for catalyst synthesis was conducted in an ^18^O-labeled solution, giving the Au/Bi^18^O_x_-TiO_2_ catalyst to perform the light-driven OCM test using ^16^O_2_ as a feeding oxygen source, as schematically illustrated in fig. S37. The produced H_2_O was then monitored by SR-PIMS (*24*). As shown in Fig. 4A, H_2_^18^O is substantially detected and its intensity gradually increases with prolonged reaction time, clearly clarifying the consumption and participation of lattice oxygen in the OCM. Such lattice oxygen-involved process can be interpreted by the upshifted O 2p band structure in BiO_x_ (Fig. 3A), which impels the thermodynamic activation of the lattice oxygen site. Under light irradiation, the photogenerated holes migrate from the TiO_2_ host to the BiO_x_ acceptor, thus activating/oxidizing the lattice oxygen ligands to electron-deficient O^(2−δ)−^ states. The consumption of lattice oxygen was further corroborated by in situ near ambient pressure XPS (NAP-XPS) measurements under the reaction gas atmosphere. As a reference, in situ XPS spectra with light irradiation were first collected under the vacuum condition. The peak intensities at lower BEs of 162.3 and 157.1 eV in Bi 4f XPS spectra are ascribed to metallic Bi species, which are substantially enhanced along with the irradiation time (fig. S38). This observation results from the lattice oxygen consumption and Bi^3+^ photoreduction induced by photogenerated electrons, which is thermodynamically feasible (*40*). When introducing CH_4_ into the XPS chamber, the Bi^3+^ photoreduction is accelerated as more metallic Bi species appear under the same irradiation time (Fig. 4B), meaning that CH_4_ can promote the lattice oxygen consumption. The irreversible loss of activated lattice oxygen in BiO_x_ leads to catalyst bleaching, as demonstrated by poor stability under very low O_2_ concentration (Fig. 2E). When O_2_ was introduced synchronously into the XPS chamber, the Bi^3+^ species were well retained under light irradiation (Fig. 4B), indicating successful chemical looping of lattice oxygen through O_2_ filling. The catalyst therefore exhibited good durability for long-term tests. To further gain underlying insight into the profound effect of O_2_, we used in situ electron spin resonance (ESR) to examine the evolution of O_2_ molecules over Au/BiO_x_-TiO_2_. Under the dark condition, the signal of OVs in recorded ESR spectra apparently reduces as O_2_ is introduced (Fig. 4C), manifesting that O_2_ rather than CH_4_ favorably fills the OV site. O_2_-TPD diagrams of BiO_x_-TiO_2_ and Au/BiO_x_-TiO_2_ samples demonstrate the broad peak located from 200° to 450°C with respect to bare TiO_2_, which is commonly assigned to the desorption of O_2_ molecules (fig. S39). The much stronger peak indicates the strong O_2_ chemisorption induced by abundant OVs in BiO_x_ NCs. The similar O_2_-TPD peak of BiO_x_-TiO_2_ and Au/BiO_x_-TiO_2_ indicates that Au does not play a great influence on O_2_ adsorption. As consolidated, the O_2_ adsorption energy (Δ*E*_O2_) was specifically calculated (fig. S40). BiO_x_ site delivers a much higher Δ*E*_O2_ value than Au site (i.e., −0.59 versus −0.24 eV), signifying the preferential O_2_ adsorption and activation over BiO_x_. Considering that the lattice oxygen in BiO_x_ NCs participates in light-driven OCM to form H_2_O, the OV sites are proposed to be refilled by O_2_. When the O_2_ concentration is relatively high, apart from the chemical looping of OV sites, the remaining O_2_ could be activated to the superoxide radical (O_2_^**·−**^) species under light irradiation, which was also experimentally corroborated by ESR measurements (see signals at *g*_xx_ = 2.021, *g*_yy_ = 2.014, and *g*_zz_ = 2.001 in Fig. 4D) (*22*, *41*, *42*). The ESR intensity of highly active O_2_^**·−**^ species reduces apparently in the presence of CH_4_, meaning that O_2_^**·−**^ species can effectively react with CH_4_. The aerobic oxidation of CH_4_ can lead to the generation of oxygenated products, thereby resulting in the overoxidation of CO_2_.

Together, the light-driven OCM reaction pathway over our advanced Au/BiO_x_-TiO_2_ catalyst is clearly proposed as follows (Fig. 4E and Eqs. 1 to 8, where O_L_ and □ represent the activated lattice oxygen site and OV in BiO_x_, respectively):

$${\text{TiO}}_{2}+h\nu \to {\text{TiO}}_{2}+e^{-}+h^{+}$$ (1)

$${\text{BiO}}_{x}+{\text{CH}}_{4}\left(g\right)\to {\text{BiO}}_{x}-{}^{*}{\text{CH}}_{4}$$ (2)

$${}^{*}{\text{CH}}_{4}+\text{Bi}-O_{L}+h^{+}\to H_{3}C^{*}-\text{Bi}-O_{L}-{}^{*}H$$ (3)

$$H_{3}C^{*}-\text{Bi}+\text{Au}\to \text{Au}-{}^{*}{\text{CH}}_{3}+\text{Bi}$$ (4)

$$H_{3}C^{*}-\text{Bi}-O_{L}-{}^{*}H+{}^{*}{\text{CH}}_{4}\to \left(H_{3}C^{*}-\text{Bi}\right)-O_{L}-\left(2^{*}H\right)$$ (5)

$$\text{Bi}-O_{L}-\left(2^{*}H\right)\to \text{Bi}-□+H_{2}O\;\left(l\right)$$ (6)

$$\text{Au}-\left(2^{*}{\text{CH}}_{3}\right)\to \text{Au}+C_{2}H_{6}\;(g)$$ (7)

$$\text{Bi}-□+1/2\;O_{2}+e^{-}\to \text{Bi}-O_{L}$$ (8)

The electrons and holes are photoexcited in the TiO_2_ photocatalyst host and migrate into the deposited BiO_x_ NCs (step 1). CH_4_ is effectively chemisorbed owing to the presence of BiO_x_ (step 2) and is easily activated to *CH_3_ and *H species through C─H dissociation at the Bi-O_L_ site with the assistance of photogenerated holes (step 3), giving rise to an intermediate state of H_3_C*-Bi-O_L_-*H. Subsequently, *CH_3_ species migrate to adjacent Au NPs (step 4), followed by further chemisorption of another CH_4_ (step 5). The activated lattice oxygen in BiO_x_ NCs binds with *H to form H_2_O, leaving the OV site (step 6). In the meantime, the *CH_3_ species that migrated to the Au site undergoes C─C coupling to produce the desired C_2_H_6_ (step 7). The OV site is refilled by an O_2_ molecule through the Mars-van Krevelen–like mechanism to achieve chemical lattice oxygen looping (step 8), balancing the reaction process. The overall reaction of OCM over our Au/BiO_x_-TiO_2_ catalyst can thus be proposed as Eq. 9

$${\text{2 CH}}_{4}\text{ + }1/2\;O_{2}\to C_{2}H_{6}+H_{2}O$$ (9)

**Fig. 5.**
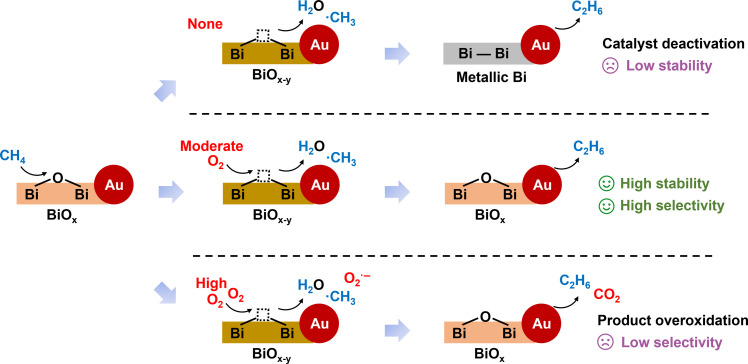
Schematic illustration of the importance of O_2_ concentration during the OCM process.

To be more specific, the evolution of introduced O_2_ during the reaction process is also clearly untangled to fundamentally interpret the underlying reaction mechanism, as concluded in Fig. 5. The critical role of O_2_ is not only to directly react with CH_4_ but also to subtly achieve the chemical looping of lattice oxygen in BiO_x_ NCs, which is governed by the O_2_ concentration. In detail, the OV site cannot be refilled timely at low O_2_ concentration, leading to the irreversible reduction of BiO_x_ to metallic Bi with catalyst deactivation. Fortunately, the formation of highly active O_2_^**·**−^ species is also avoided under a low O_2_ concentration scenario. Therefore, low activity and poor stability but high selectivity for C_2_H_6_ products are observed. Increasing the O_2_ concentration can not only promote the lattice oxygen looping process through a Mars-van Krevelen–like pathway to improve the activity but also retain the states of oxidized Bi^3+^ species for stability, thus contributing to high activity, high stability, and high selectivity at a moderate O_2_ concentration (0.05 vol % in our study). Nevertheless, a further increase in O_2_ concentration will give rise to the formation of O_2_^−^ species that unfavorably overoxidize the intermediates during the OCM process, leading to poor selectivity for C_2_H_6_ products.

## DISCUSSION

In summary, we have demonstrated an integrated Au/BiO_x_-TiO_2_ hybrid photocatalyst to achieve a highly efficient light-driven OCM reaction with C_2_H_6_ as the dominant product. The intrinsic bottlenecks of OCM, i.e., low activity and selectivity, are well addressed by virtue of rational catalytic site engineering for CH_4_ activation and C─C coupling. Specifically, the upshifted band structure in BiO_x_ facilitates CH_4_ chemisorption and turns on the lattice oxygen activation to promote the pivotal C─H dissociation. In addition, Au guides the transfer of dissociated *CH_3_ species from the Bi site to undergo the following C─C coupling, avoiding undesirable overoxidation. The participation of activated lattice oxygen in the OCM reaction contributes to the formation of H_2_O, whose remaining OVs are chemically looped through the Mars-van Krevelen pathway under the appropriate O_2_ concentration, endowing good catalytic stability. As a result, an optimal Au/BiO_x_-TiO_2_ hybrid delivers a remarkable CH_4_ conversion rate of 20.8 mmol g^−1^ hour^−1^, along with a high C_2+_ product selectivity of 97% and a specific C_2_H_6_ production rate of 9.6 mmol g^−1^ hour^−1^. The OCM can be stably operated for up to 50 hours in a flow reactor. This work underscores the importance of catalytic site engineering for chemical reaction manipulation and opens a promising avenue toward sustainable and energy-efficient CH_4_ conversion.

## MATERIALS AND METHODS

### Chemicals

All the chemical reagents were used without further purification. Tetrabutyl titanate (C_16_H_36_O_4_Ti, TBOT; 97%) and chloroauric acid (HAuCl_4_·4H_2_O; 99.9%) were obtained from Sigma-Aldrich. Hydrofluoric acid (HF; 30% in H_2_O) and bismuth nitrate pentahydrate [Bi(NO_3_)_3_·5H_2_O; 99.9%] were purchased from Sinopharm. The water used in all experiments was deionized.

### Synthesis of TiO_2_ nanosheets substrate

TiO_2_ nanosheets were prepared by the hydrothermal method. In detail, 10 ml of TBOT was mixed with 1.6 ml of HF. Then, the mixed solution was transferred into a Teflon-lined autoclave and heated at 180°C for 24 hours. The resultant product was separated by centrifugation, washed with water and anhydrous ethanol several times, and then dried at 60°C in a vacuum oven.

### Synthesis of BiO_x_-TiO_2_ samples

Two hundred milligrams of TiO_2_ nanosheets was first dispersed into 50 ml of H_2_O with vigorous stirring for 30 min. Then, 12-mg Bi(NO_3_)_3_·5H_2_O was added to the solution. The mixed solution was bubbled with Ar for 30 min to eliminate the dissolved O_2_. The suspension was irradiated by a 300-W xenon lamp for 1 hour under an Ar atmosphere. After that, the collected solid product was calcined in air at 450°C for 2 hours to obtain the 2.5-BiO_x_-TiO_2_ sample (named BiO_x_-TiO_2_ in the manuscript), where 2.5 is the Bi loading amount determined by ICP measurement. 1.2-BiO_x_-TiO_2_ and 5-BiO_x_-TiO_2_ samples were synthesized by the same method except changing the Bi(NO_3_)_3_·5H_2_O amount by 6 and 24 mg, respectively.

### Synthesis of Au/BiO_x_-TiO_2_ samples

One hundred milligrams of as-obtained BiO_x_-TiO_2_ was first dispersed into 50 ml of H_2_O. One hundred eighty-two microliters of HAuCl_4_ solution (2.7 mg ml^−1^) was attenuated with H_2_O solution to 10 ml, which was slowly injected into the BiO_x_-TiO_2_ suspension with stirring for 1 hour. Then, the mixed solution was bubbled with Ar for 30 min to eliminate dissolved O_2_. The suspension was irradiated by a 300-W xenon lamp for 15 min. Last, the product was washed with water several times and dried at 60°C in a vacuum oven.

### Characterizations

Powder XRD patterns were recorded by using a Philips X’Pert Pro Super x-ray diffractometer with Cu Kα radiation (λ = 1.54178 Å). TEM images were taken on a Hitachi Model H-7700 microscope at 100 kV. High-resolution TEM images were recorded on an FEI Talos F200X field-emission high-resolution transmission electron microscope at 200 kV. HAADF-STEM images and EDS mapping profiles were recorded on a JEOL ARM-200F atomic resolution TEM. XPS spectra were collected on a Thermo Scientific Escalab 250Xi x-ray photoelectron spectrometer using a non-monochromatized Al-Kα x-ray (1486.6 eV) as the excitation source. UV-Vis diffuse reflectance spectra were recorded in the spectral region of 200 to 800 nm with a Shimadzu SolidSpec-3700 spectrophotometer. EPR spectra were recorded by a JEOL JES-FA200 ESR spectrometer (9.062 GHz). CH_4_-TPD and O_2_-TPD were recorded by using an AutoChem II 2920 (Micromeritics Instrument Corporation). Catalysts were pretreated in a He atmosphere at a flow rate of 20 ml min^−1^ for 60 min at 200°C. After cooling to room temperature, adsorption was performed under a pure CH_4_ or O_2_ atmosphere for 90 min. Then, CH_4_-TPD and O_2_-TPD were recorded at a heating rate of 10°C min^−1^ with the carrier gas He. The Au and Bi elemental content analysis of the samples was carried out by ICP-MS (PerkinElmer, NexION 5000). XAFS spectra of Bi L_3_-edge were acquired in fluorescence mode at the beamline BL14W1 in the Shanghai Synchrotron Radiation Facility. The EXAFS data were processed according to the standard procedure Athena program implemented in the IFEFFIT software packages.

### In situ DRIFTS measurements

In situ DRIFTS measurements were performed on a Bruker IFS 66 v Fourier-transform spectrometer equipped with Harrick diffuse reflectance accessory at the beamline BL01B in the National Synchrotron Radiation Laboratory (NSRL). The sample was mixed with KBr and loaded on a customized photocatalytic reaction cell. After sample loading, pure Ar was introduced into the system for 30 min to collect background spectra. Then, pure CH_4_ was bubbled into the system and the spectra were recorded in a CH_4_ atmosphere. After that, the spectra were recorded with light irradiation at 1, 5, 10, 20, and 30 min, respectively.

### In situ SR-PIMS measurements

In situ SR-PIMS measurements were performed at the beamline BL03U in NSRL. The samples were loaded into quartz glass. After sample loading, the quartz glass was connected to a photoionization mass spectrometer and pumped into the vacuum. Then, CH_4_ was introduced into the glass at a 20-sccm rate for 30 min. After that, the sample was illuminated by a 300-W Xe lamp, and the signal was detected at a photoenergy of 10.3 eV with light irradiation at 0, 300, 600, 900, and 1200 s.

### In situ NAP-XPS measurements

NAP-XPS spectra were collected at SPECS NAP-XPS equipment. Typically, the sample was dispersed on a silicon wafer and loaded in a vacuum chamber overnight. First, the XPS spectra were recorded under vacuum. Then, CH_4_ gas was purged into a chamber at a pressure of up to 100 Pa. The spectra were recorded under dark and light irradiation conditions, respectively. After that, CH_4_ and O_2_ gasses were purged into a chamber with pressure up to 100 Pa, and the spectra were recorded in the same way.

### Photocatalytic OCM experiments

Photocatalytic OCM experiments were carried out at room temperature and atmospheric pressure in a quartz flow reactor (as shown in fig. S15). Typically, 5 mg of catalyst was dispersed on a glass fiber membrane. Then, pure CH_4_ and a mixed gas of Ar/O_2_ were purged into a reactor with a total flow rate of 71 ml min^−1^ (gas ratio CH_4_/mixed gas, 70/1) for 30 min by mixture gas control assembly, wherein the specific O_2_ concentration was determined by gas chromatography (GC). The O_2_ concentration in feeding gas was adjusted by changing the ratio of CH_4_/mixed gas. After that, the reactor was irradiated by a 300-W xenon lamp (PLS-SXE300, Perfect light) with a light intensity of 600 mW cm^−2^. The gas products were detected by a GC (GC-2014 + AFSC, Shimadzu) equipped with a methanation reactor, flame ionization detector, and thermal conductivity detector. The product selectivity was calculated according to the observable products (C_2_H_6_, C_3_H_8_, and CO_2_), and the related equation is shown below

$$C_{2+}\;\text{Selectivity}=\frac{n_{C_{2}H_{6}}\times 2+n_{C_{3}H_{8}}\times 3}{n_{C_{2}H_{6}}\times 2+n_{C_{3}H_{8}}\times 3+n_{{\text{CO}}_{2}}}\times 100\%$$

where *n_x_* is the amount of the detected products (*x* = C_2_H_6_, C_3_H_8_, and CO_2_). The AQE was obtained by using different monochromatic filters (365, 380, 400, and 500 nm) during light irradiation and was calculated according to the following equation

$$\text{AQE}=\frac{N_{\text{electron}}}{N_{\text{photon}}}\times 100\%=\frac{n\times n_{C_{2}H_{6}}\times N_{A}}{\frac{W\times S\times t}{h\times \nu }}\times 100\%$$

where *N*_electron_ and *N*_photon_ represent the number of reacted electrons and incident photons, respectively, *n* is the number of transferred electrons for C_2_H_6_ formation, *n*_C_2_H_6__ is the molar number of C_2_H_6_, N_A_ is the Avogadro’s constant, and *W*, *S*, *t*, *h*, and *ν* are the light intensity, irradiation area, irradiation time, Planck constant, and light frequency, respectively. The TON of C_2_H_6_ product after the stability test was calculated by the equation of

$$\text{TON}=\frac{n_{C_{2}H_{6}}\times 2}{n_{\text{site}}}$$

where *n*_C_2_H_6__ is the total molar number of produced C_2_H_6_, and *n*_site_ is the loading molar number of Bi or Au active site.

### Isotope-labeling experiments

The ^13^C-isotope labeling experiments were performed by using pure ^13^CH_4_ as the feeding gas. The C_2_H_6_ product was analyzed using gas chromatography MS (GC-MS, 7890A-5975C, He carrier, Agilent). To trace the oxygen atom of the H_2_O product, the BiO_x_ NCs deposited on TiO_2_ were labeled by ^18^O_2_ during the synthetic process. First, 100-mg TiO_2_ nanosheets were dispersed into 50 ml of ^18^O-labeled H_2_^18^O with vigorous stirring for 30 min. Then, 6-mg Bi(NO_3_)_3_·5H_2_O and 182-μl HAuCl_4_ solution (2.7 mg ml^−1^) were added into the solution. The mixed solution was bubbled with Ar for 30 min to eliminate dissolved O_2_. The suspension was irradiated by a 300-W xenon lamp for 60 min. After that, ^18^O-labeled ^18^O_2_ was further bubbled into the suspension until the color changed from black to yellow. The photo-deposited BiO_x_ NCs were thus ^18^O-labeled. Then, to trace the oxygen atom of product H_2_O, the product H_2_O was recorded by in situ SR-PIMS (beamline BL03U of NSRL).

### Computational details

Theoretical calculations were performed using density functional theory as implemented in the VASP code with exchange-correlation energy functional, which were modeled by Perdew-Burke-Ernzerhof functional (*43*–*45*). A Monkhorst-Pack grid of 9 × 9 × 4 *k*-points was used to sample the Brillouin zone of the unit cell of anatase TiO_2_. A (3 × 3) supercell with four layers of TiO_2_ was used to describe the (001) surface. The thickness of the vacuum in all slabs was set to 20 Å to eliminate the interactions between the layers caused by the periodic boundary condition. The cutoff energy was set to 520 eV and structural relaxation was conducted with the criteria of energy convergence of 10^−5^ eV per atom and force convergence of 0.01 eV/Å, respectively. The vdW-DF2 method was applied to describe the long-range van der Waals (vdW) interactions in all the structures (*46*, *47*). According to experimental results, a computational model of a TiO_2_ (100) surface that supported Bi_2_O_3_ NC was used to investigate the CH_4_ adsorption and dissociation behaviors. Meanwhile, a model of an Au (111) surface–supported Bi_2_O_3_ NC was constructed to evaluate the migration energy of ·CH_3_ from the Bi_2_O_3_ cluster to the Au surface. The adsorption energies (Δ*G*_ads_) of CH_4_ were defined as Δ*G*_ads_ = *G*_total_ − *E*_surf_ − *G*_CH_4__, where *G*_total_, *E*_surf_, and *G*_CH_4__ represent the energies of adsorption configurations, computational surfaces, and CH_4_, respectively. All the energies were corrected by considering the zero-point energy and entropy corrections under standard conditions (*p*_0_ = 1 bar and *T*_0_ = 298.15 K). Then, two sequential deprotonation steps were considered to depict the dissociation process of CH_4_ on TiO_2_ and BiO_x_/TiO_2_ surfaces, respectively.

$${{\text{CH}}_{4}}^{*}\to {{\text{CH}}_{3}}^{*}+H^{+}+e^{-}$$ (Step 1)

$${{\text{CH}}_{3}}^{*}\to {{\text{CH}}_{2}}^{*}+H^{+}+e^{-}$$ (Step 2)

Note that the chemical potentials of H^+^ and e^−^ can be described based on the computational hydrogen electrode (*48*). Thus, the dissociation energy of the above steps (Δ*G*) can be calculated via the following equations

$$\Delta G_{1}=G_{{}^{*}{\text{CH}}_{\text{3}}}+G_{{}^{*}H}-G_{{}^{*}{\text{CH}}_{\text{4}}}$$

$$\Delta G_{2}=G_{{}^{*}{\text{CH}}_{2}}+G_{{}^{*}H}-G_{{}^{*}{\text{CH}}_{3}}$$

## Supplementary Materials

### This PDF file includes:

Figs. S1 to S42 Tables S1 to S4 Note S1 References
