# Supplementary material for: Highly efficient, selective, and stable photocatalytic methane coupling to ethane enabled by lattice oxygen looping
Source: Sci Adv. 2024 Jun 28;10(26):eado4390. doi: 10.1126/sciadv.ado4390 (PMC11637002; doi:10.1126/sciadv.ado4390)
Supplement: Supplementary file 1 — Figs. S1 to S42 Tables S1 to S4 Note S1 References [file sciadv.ado4390_sm.pdf]

Science Advances
AAAS

Supplementary Materials for

Highly efficient, selective, and stable photocatalytic methane coupling to ethane enabled by lattice oxygen looping

Guangyao Zhai *et al.*

Corresponding author: Ning Zhang, zhangning18@ustc.edu.cn; Dong Liu, dongliu@ustc.edu.cn; Yujie Xiong, yjxiong@ustc.edu.cn

Sci. Adv. **10**, eado4390 (2024)
DOI: 10.1126/sciadv.ado4390

This PDF file includes:

Figs. S1 to S42
Tables S1 to S4
Note S1
References

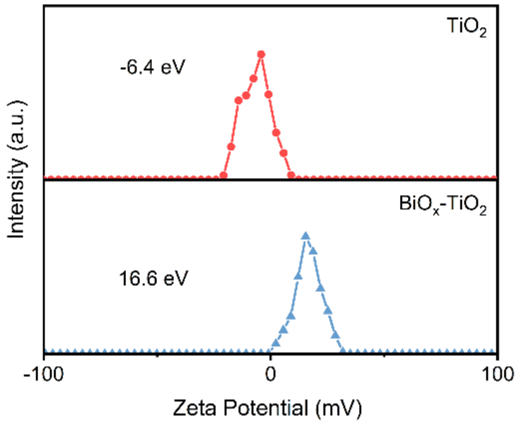

Fig. S1. Zeta potential of pure TiO_2 and BiO_x-TiO_2.

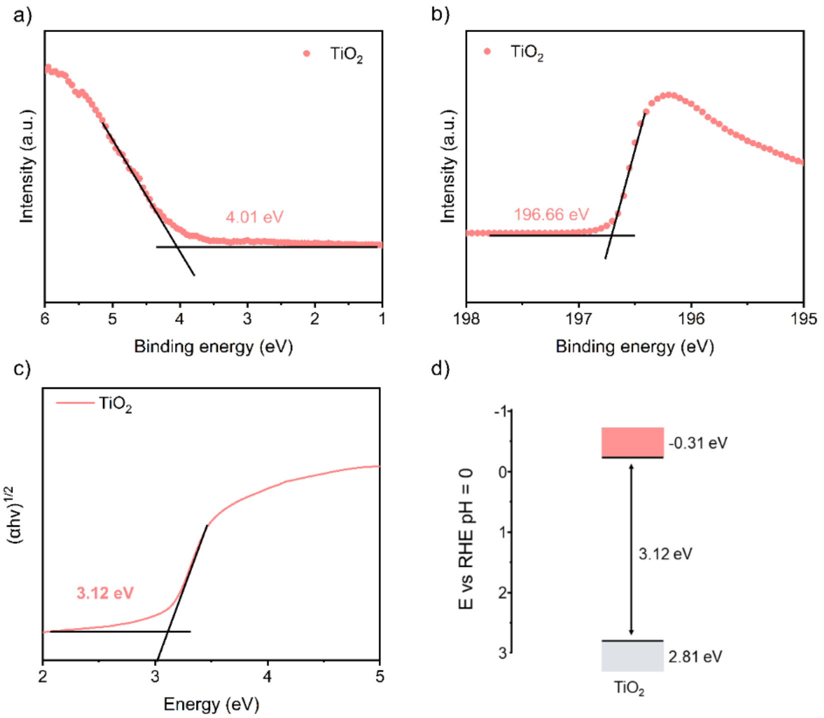

Fig. S2. Energy band structure of TiO_2. (a) Valence band (VB) spectra, (b) secondary electron cutoff (E_{cutoff}) spectra, (c) bandgap determination through Tacu plot, and (d) the calculated energy band structure versus RHE.

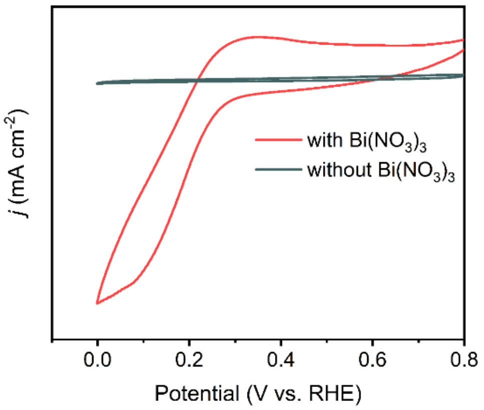

Fig. S3. Electrochemical tests of redox potential. The measured redox potential of Bi^{3+}/Bi^{0} through electrochemical CV scanning.

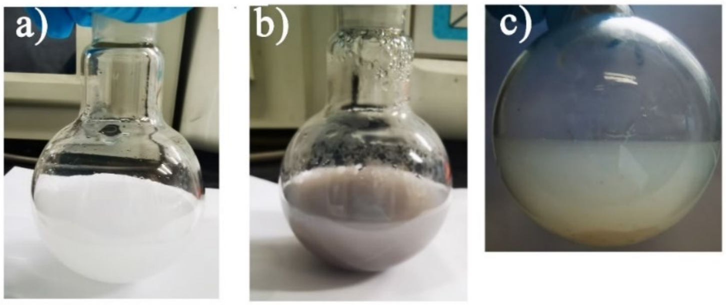

Fig. S4. The change in color of solution during the synthesis process. (a) before reaction under Ar atmosphere (white), (b) after photo reduction reaction under Ar atmosphere (black), and (c) after exposure to the air (yellow).

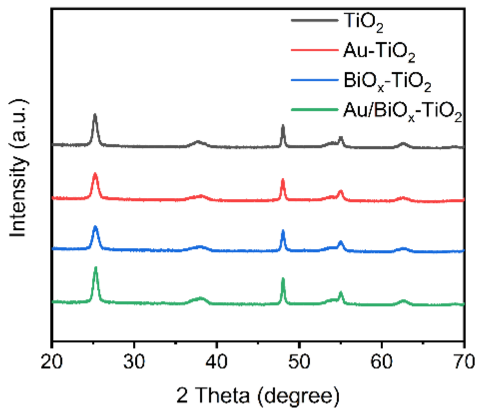

Fig. S5. Structure characterization. XRD patterns of as-synthesized TiO_2, Au-TiO_2, BiO_x-TiO_2 and Au/BiO_x-TiO_2 samples.

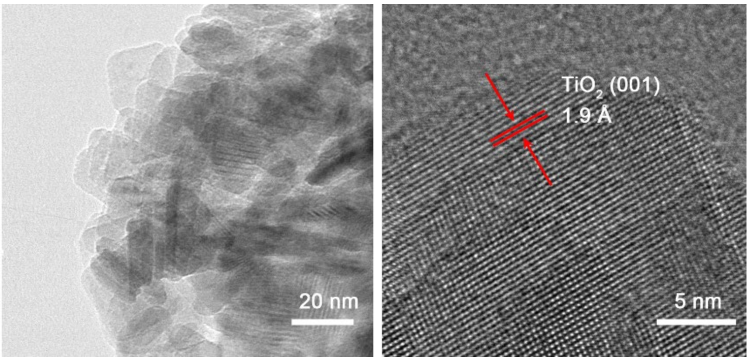

Fig. S6. TEM images of TiO_2 substrate.

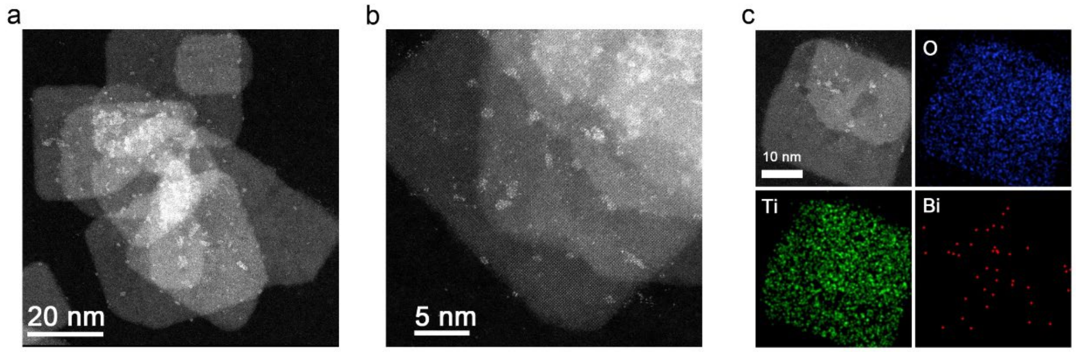

Fig. S7. Atomic-resolution electron microscopy characterization. (a, b) HAADF-STEM image of BiO_x-TiO_2 sample and (c) the related elemental mapping with O (blue), Ti (green), and Bi (red) elements.

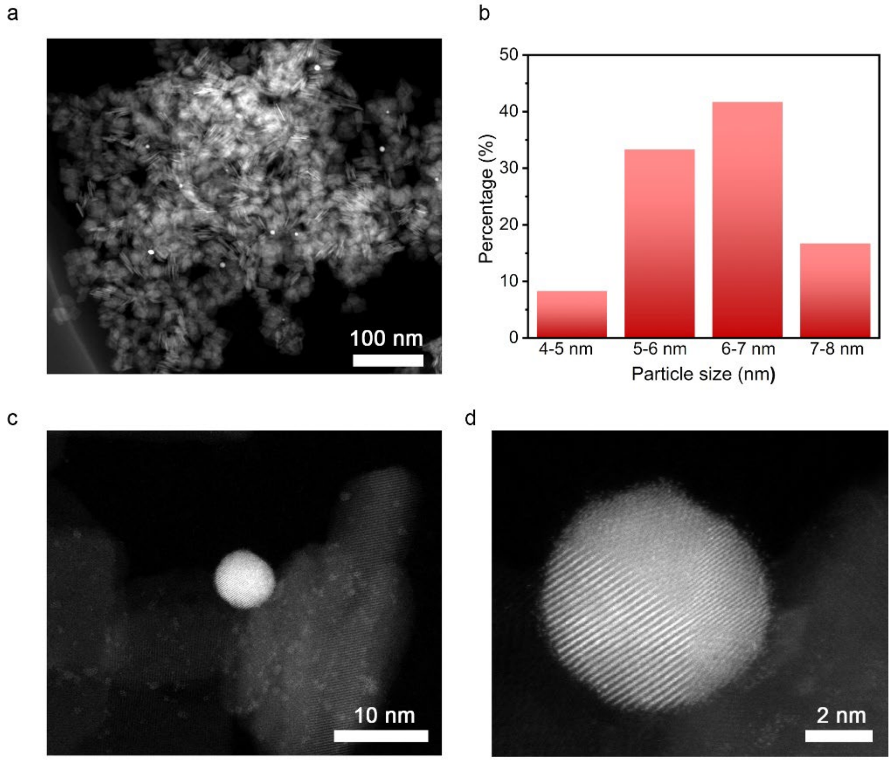

Fig. S8. Morphology characterizations. (a) TEM image of Au/BiO_x-TiO_2, (b) size distribution of Au nanoparticles of Au/BiO_x-TiO_2, and (c, d) HAADF-STEM images of Au/BiO_x-TiO_2.

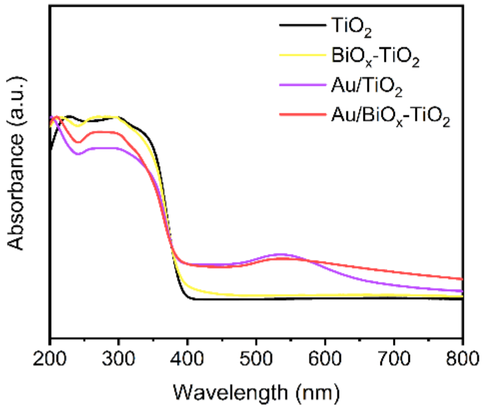

Fig. S9. UV–vis absorption spectra of TiO_2, Au-TiO_2, BiO_x-TiO_2 and Au/BiO_x-TiO_2.

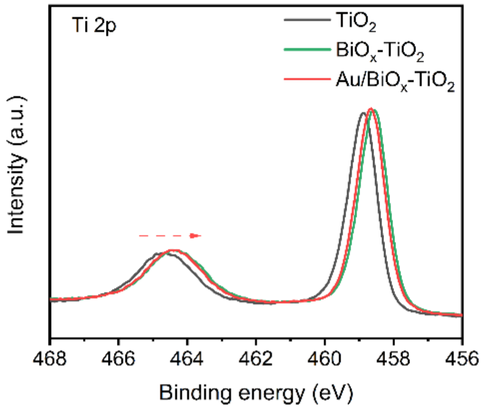

Fig. S10. High-resolution Ti 2p XPS spectra of TiO_2, BiO_x-TiO_2, and Au/BiO_x-TiO_2.

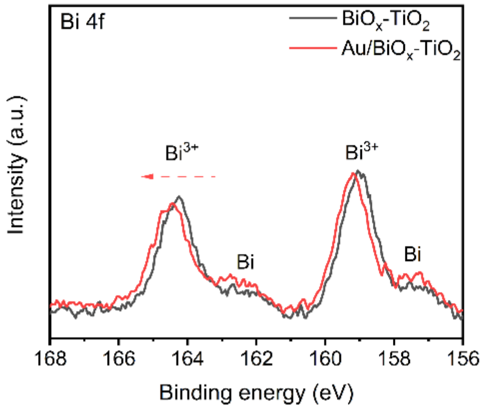

Fig. S11. High-resolution Bi 4f XPS spectra of BiO_x-TiO_2 and Au/BiO_x-TiO_2.

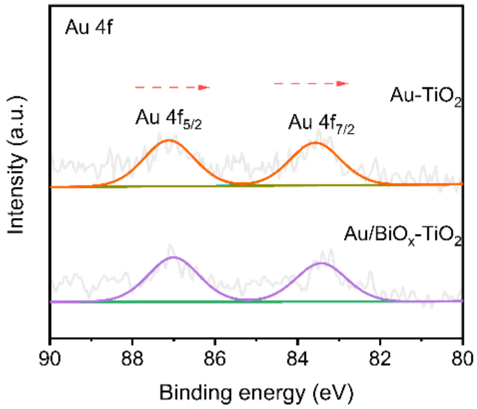

Fig. S12. High-resolution of Au 4f XPS spectra of Au-TiO_2 and Au/BiO_x-TiO_2.

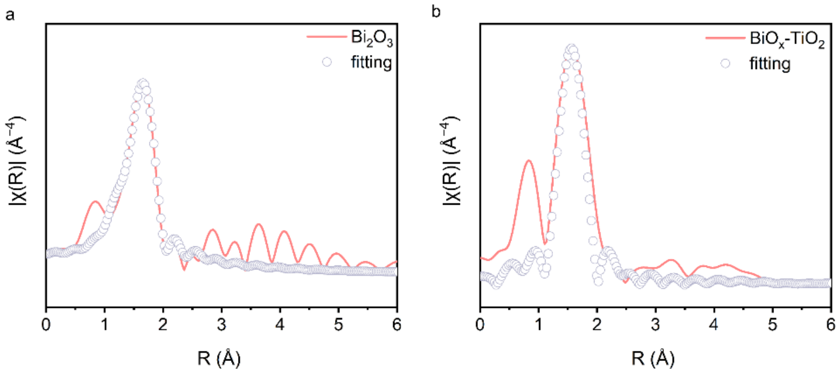

Fig. S13. XANES characterization. Fourier-transformed Bi L_3-edge EXAFS spectra and the related fitting curve of (a) Bi_2O_3 and (b) BiO_x-TiO_2.

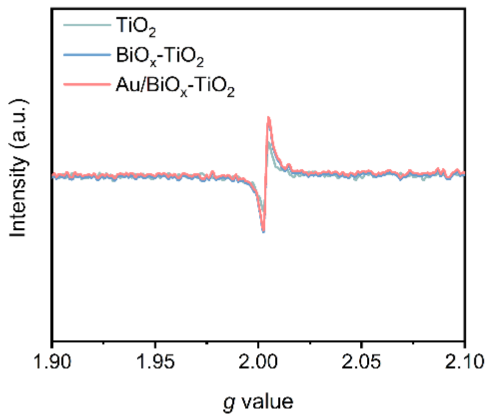

Fig. S14. EPR spectra of TiO_2, BiO_x-TiO_2 and Au/BiO_x-TiO_2 samples.

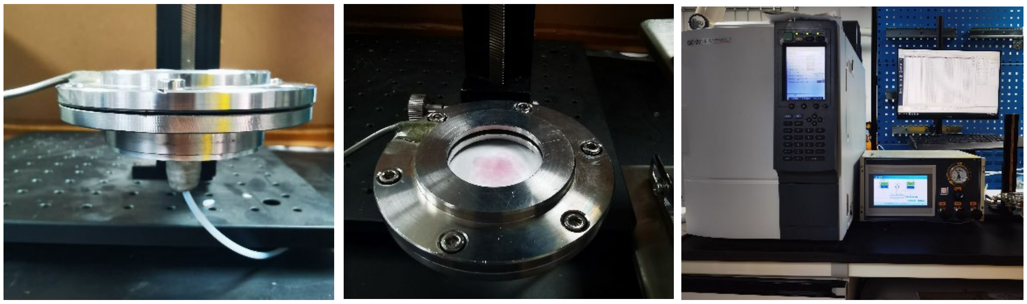

Fig. S15. Setup of flow reactor system for photocatalytic CH_4 conversion.

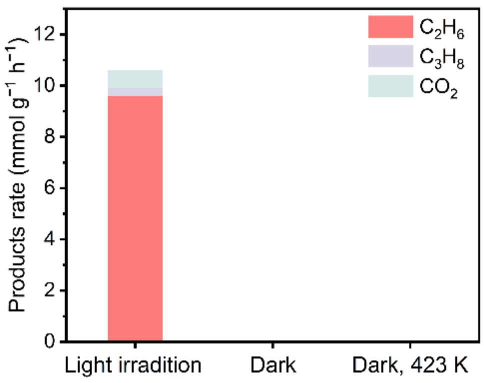

Fig. S16. OCM performance over Au/BiO_x-TiO_2 under different reaction conditions.

Reaction condition: at room temperature or with extra 423K heating.

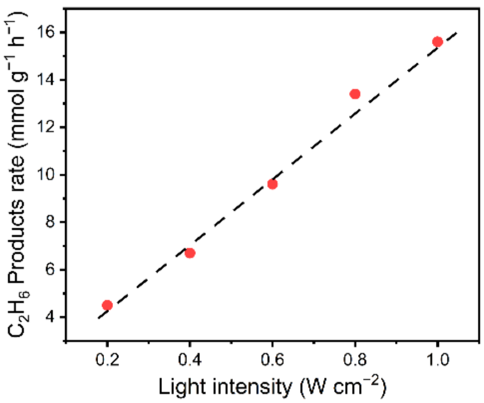

Fig. S17. C_2H_6 production rate of OCM performance over Au/BiO_x-TiO_2 under different light intensity.

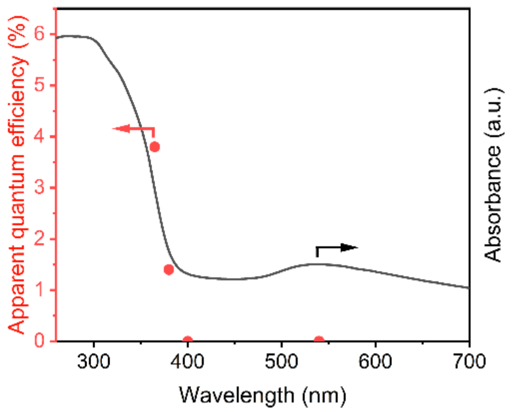

Fig. S18. AQE measurements. Wavelength dependence of AQE over the Au/BiO_x-TiO_2 catalyst together with UV–vis diffuse reflectance spectrum.

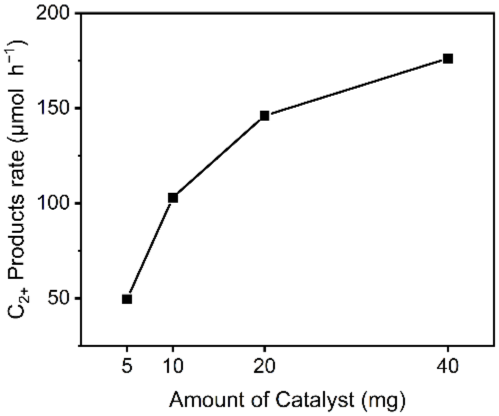

Fig. S19. Photocatalytic OCM performance of Au/BiO_x-TiO_2 with difference catalyst dosage.

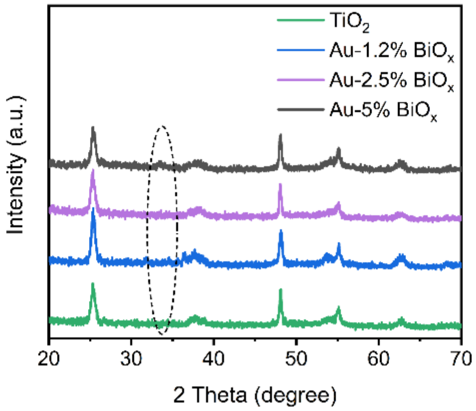

Fig. S20. XRD characterization. XRD pattern of TiO_2, Au-1.2% BiO_x, Au-2.5% BiO_x, Au-5% BiO_x samples.

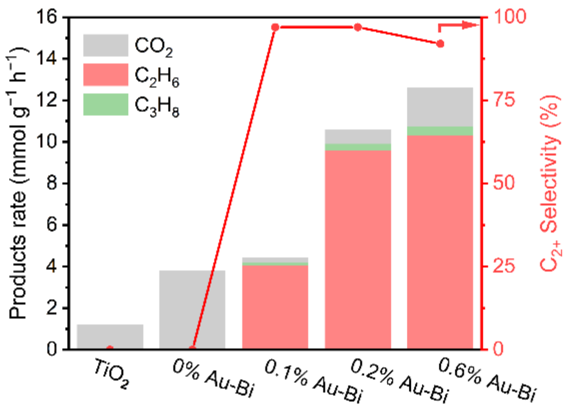

Fig. S21. Photocatalytic OCM performance. Photocatalytic activity and selectivity of C_{2+} products over Au/BiO_x-TiO_2 catalysts with different Au loading amounts. (The Bi loading amount is fixed to be 2.5 wt%.)

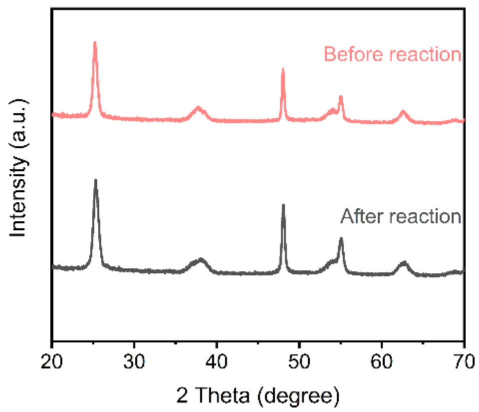

Fig. S22. XRD patterns of Au/BiO$_x$-TiO$_2$ before and after reaction.

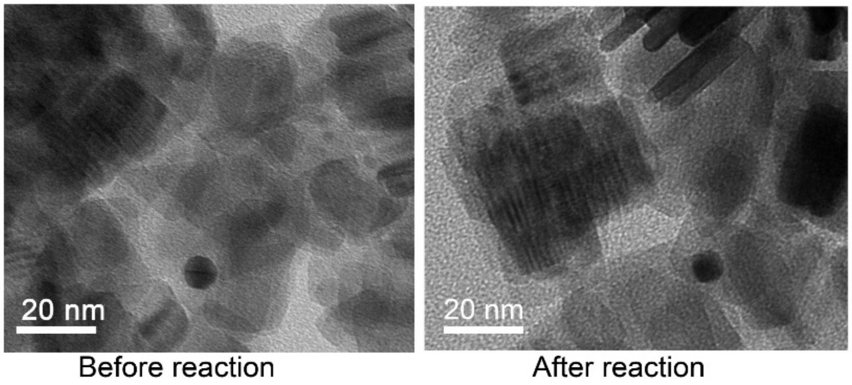

Fig. S23. TEM images of Au/BiO_x-TiO_2 before and after reaction.

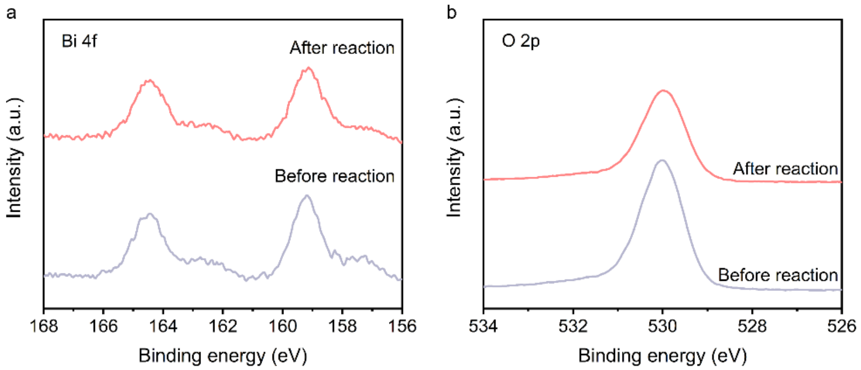

Fig. S24. XPS characterization. (a) Bi 4f XPS spectra and (b) O 2p XPS spectra over Au/BiO_x-TiO_2 before and after reaction.

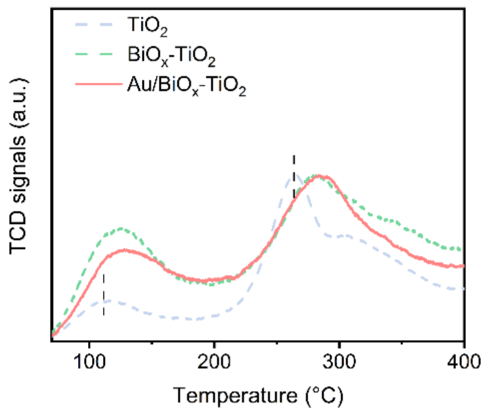

Fig. S25. **CH_4-TPD spectra of TiO_2, BiO_x-TiO_2 and Au/BiO_x-TiO_2.**

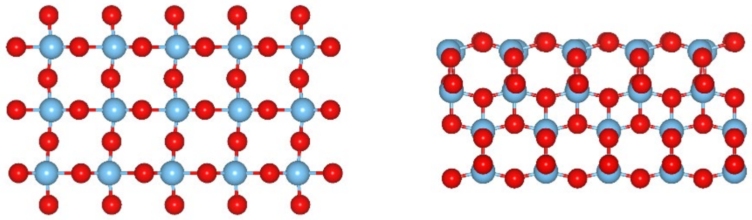

Fig. S26. The schematic illustration of simulated TiO_2 model.

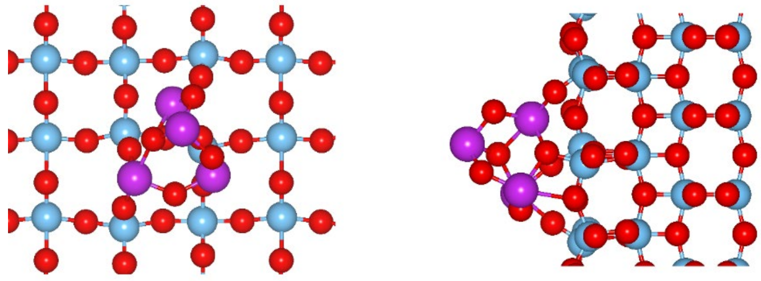

Fig. S27. The schematic illustration of simulated BiO_x-TiO_2 model.

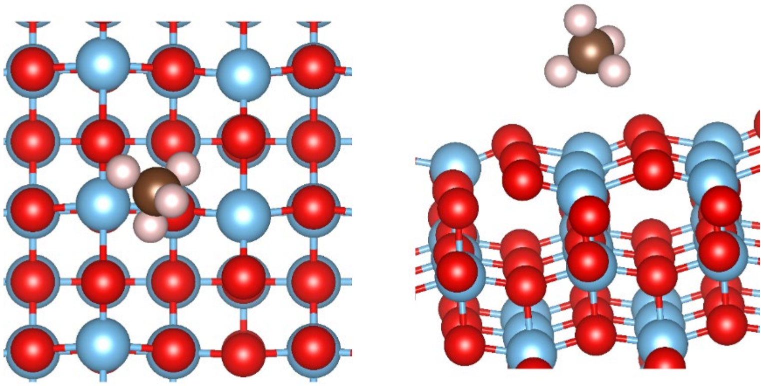

Fig. S28. The schematic illustration of CH_4 adsorption on TiO_2 model.

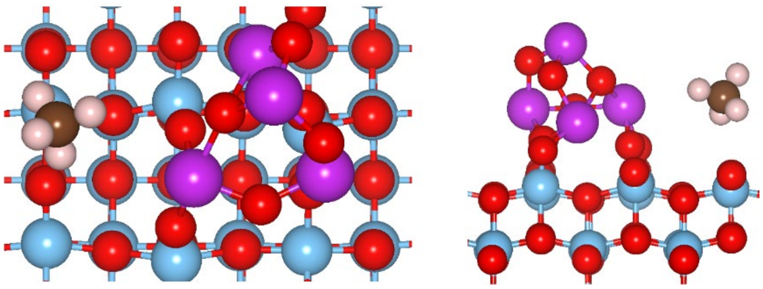

Fig. S29. The schematic illustration of CH_4 adsorption on BiO_x-TiO_2 model.

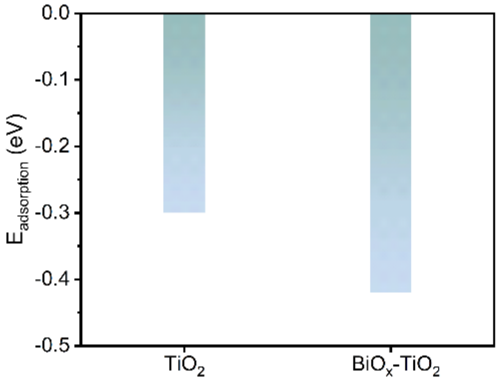

Fig. S30. The calculated CH_4 adsorption energies over TiO_2 and BiO_x-TiO_2 models.

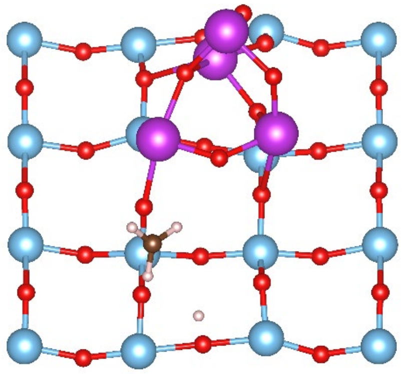

Fig. S31. The schematic illustration of transition state for first C–H dissociation of CH_4 on TiO_2 model.

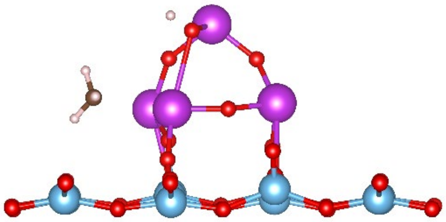

Fig. S32. The schematic illustration of transition state for first C–H dissociation of CH_4 on BiO_x model.

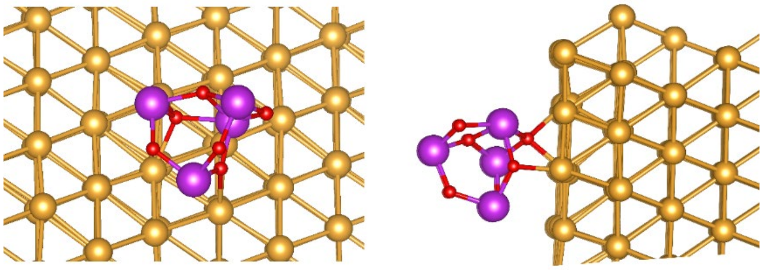

Fig. S33. The schematic illustration of simulated Au-BiO$_x$ model.

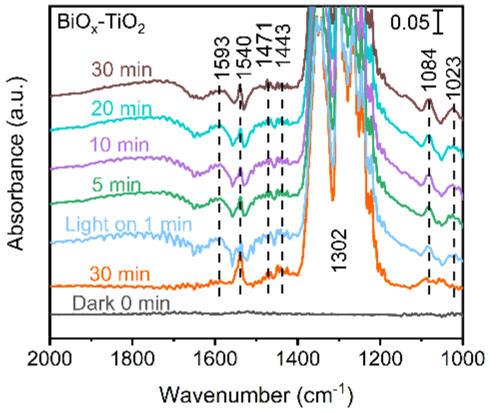

Fig. S34. *In situ* DRIFTS characterizations. *In situ* DRIFTS spectra for photocatalytic conversion of CH_4 with different time over BiO_x-TiO_2.

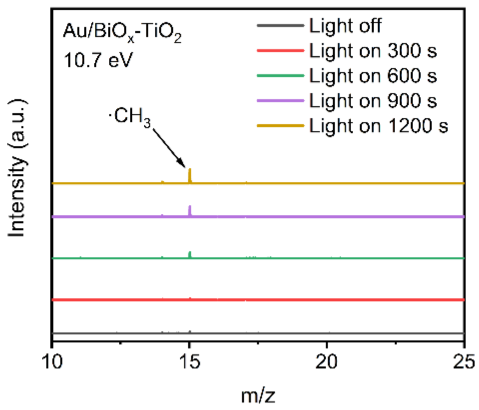

Fig. S35. *In situ* SR-PIMS spectra. *In situ* SR-PIMS of $\cdot CH_3$ radical signal over $Au/BiO_x\text{-}TiO_2$ at photon energy of 10.7 eV with different illumination time.

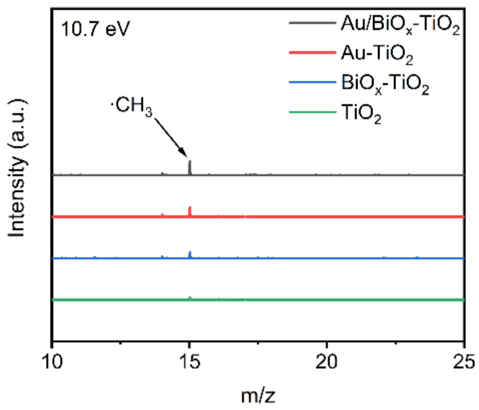

Fig. S36. *In situ* SR-PIMS spectra. *In situ* SR-PIMS spectra $\cdot CH_3$ radical signal over TiO_2, BiO_x-TiO_2, Au-TiO_2 and Au/BiO_x-TiO_2 at photon energy of 10.7 eV with the light irradiation of 1200 s.

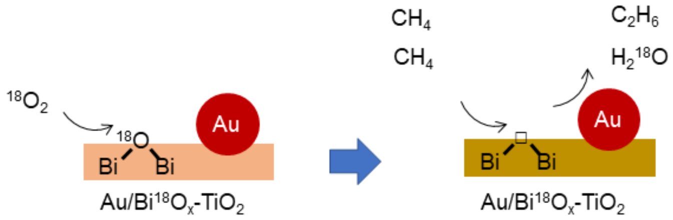

Fig. S37. The schematic illustration·of.^{18}O-abelled experiments. The ^{18}O labelled experiments are carried out to investigate whether lattice oxygen participates into the reaction or not.

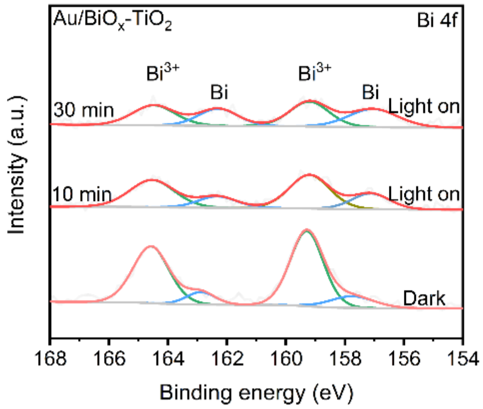

Fig. S38. *In situ* XPS spectra. *In situ* XPS spectra of Bi 4f spectra over Au/BiO_x-TiO_2 under the vacuum condition with different light irradiation time.

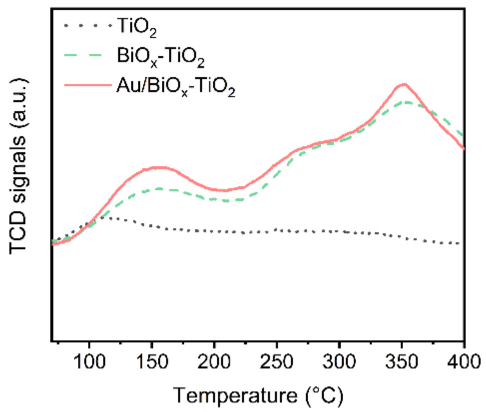

Fig. S39. O_2-TPD spectra of TiO_2, BiO_x-TiO_2 and Au/BiO_x-TiO_2.

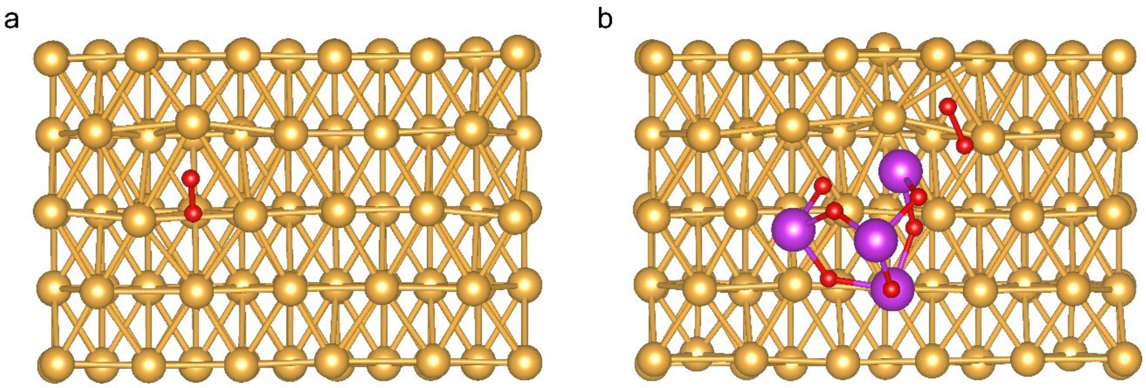

Fig. S40. O_2 adsorption model. The schematic illustration of O_2 adsorption on (a) Au and (b) BiO_x sites, respectively.

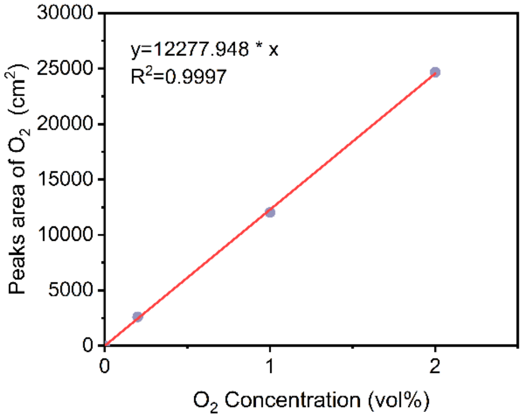

Fig. S41. Calibration curve for quantification of O_2 by GC measurement.

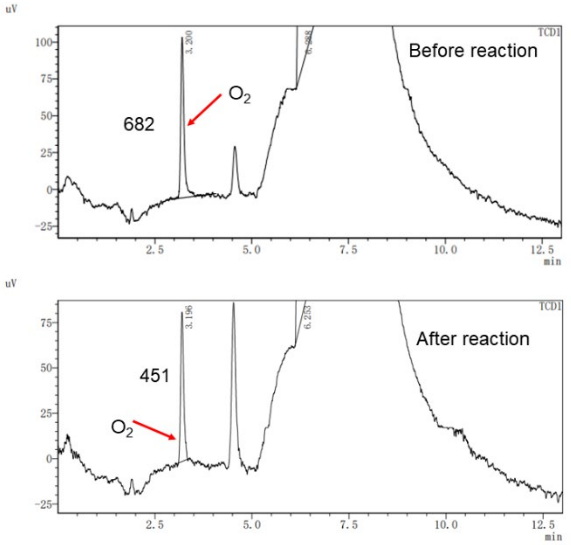

Fig. S42. Gas product analysis. The peaks area of O_2 before and after reaction detected by GC with thermal conductivity detector (TCD).

Table S1. The determined contents of Bi and Au in different samples.

Sample	Content of Bi	Content of Au
Au-1.2 % Bi	1.2 wt.%	0.2 wt.%
Au-1.2 % Bi	2.4 wt.%	0.2 wt.%
Au-1.2 % Bi	4.8 wt.%	0.2 wt.%
0.1% Au-Bi	2.4 wt.%	0.09 wt.%
0.2% Au-Bi	2.4 wt.%	0.18 wt.%
0.6% Au-Bi	2.4 wt.%	0.55 wt.%

Table S2. Fitting results of Bi L_3-edge EXAFS data of BiO_x-TiO_2 and Bi_2O_3 reference.

Sample	Path	CN	R (Å)	σ^2 (Å)	ΔE_0 (eV)
BiO_x-TiO_2	Bi-O	2.2±0.5	2.16±0.01	0.005±0.003	-2.16±2.97
Bi_2O_3	Bi-O	3 (fixed)	2.14±0.01	0.004±0.001	-1.28±1.67

CN is the coordination number, R is the bonding distance, σ^2 is the Debye-Waller factor, and ΔE_0 is the edge-energy shift.

Table S3. The TONs of photocatalytic methane conversion to C_2 product for representative works.

Entry	Catalysts	Mass (mg)	Metal amount (wt%)	Time (h)	C_2 Yield (mmol/g/h)^b	TON	Ref.
**1**	**Au/BiO_x-TiO_2**	**5**	**0.2 for Au**<br>**2.5 for Bi**	**50**	**9.6**	**94675**<br>**17897**	**This work**
2	Au-TiO_2	20	2.9	30	55	22417	(*4*)
3	Ag-HPW/TiO_2	100	0.6	7	0.016	4	(*7*)
4	Au/ZnO-TiO_2	5	1	12	5	2364	(*9*)
5	CuO_x/Pt-PC50	100	0.1	8	0.0068	10	(*10*)
6	ZnO-$AuPd_{2.7\%}$	2	2.7	8	0.067	47	(*15*)
7	CuPd-TiO_2	50	0.08	112	2.2	12642	(*23*)
8	Pd-TiO_2	3	0.2	24	0.9	3816	(*31*)
9	Au-TiO_2	5	2	240	16.4	77539	(*49*)

Table S4. Selective recent reports of photocatalytic CH_4 conversion to C_{2+} products.

Catalyst	Reaction conditions	C_2H_6 rate (μmol/g/h)	C_{2+} selectivity (%)	Stability (h)	Ref.
**Au/BiO_x-TiO_2**	**300 W Xe lamp (400 mW cm^{-2}); 5-mg catalyst**	**~9600**	**97**	**50**	**This work**
**Au/BiO_x-TiO_2**	**300 W Xe lamp (1 W cm^{-2}); 5-mg catalyst**	**~15400**	**95**	**/**	**This work**
Au-TiO_2	100 W LED (365 nm); 393K; 20-mg catalyst	55000	90	30	(*4*)
Nb-TiO_2-SiO_2	300 W Xe lamp; 100-mg catalyst	~1.69	96	16	(*5*)
Pt/Ga-TiO_2-SiO_2	300 W Xe lamp; 200-mg catalyst	~1.68	90	32	(*6*)
Ag-HPW/TiO_2	400 W Xe lamp; 100-mg	~20.6	90	7	(*7*)
Ag-AgBr-TiO_2	LED (365 nm) 100-mg catalyst	354	79	12	(*8*)
Au-ZnO/TiO_2	300 W Xe lamp; 20-mg catalyst	~5000	90	12	(*9*)
$Cu_{0.1}Pt_{0.5}$/PC-50	40 W LED (365 nm); 100-mg catalyst	~65	60	8	(*10*)
Zn-ZSM-5	150 W Hg lamp; 1-g catalyst	~2.985	99	8	(*13*)
ZnO-$AuPd_{2.7}$	300 W Xe lamp; 2-mg catalyst	~39.3	96	10	(*15*)
Au/ZnO	LED (365 nm) 20-mg catalyst	6837	83	48	(*22*)
PdCu/TiO_2	40 W LED (365 nm); 50-mg catalyst	2480	57	112	(*23*)
$Au_{0.05}Pd_{0.05}/TiO_2$	300 W Xe lamp; 10-mg catalyst	10092	77	32	(*26*)
Pd_1/TiO_2	300 W Xe lamp; 3-mg catalyst	~913	94	6	(*31*)

Note S1. The calculation of oxygen balance during OCM.

We determined the consumption of oxygen in the feeding gas through GC measurements to assess the oxygen balance in our flow reactor, which can be calculated according to the following formulas:

$$2CH_4 + 1/2O_2 \rightarrow C_2H_6 + H_2O$$

$$3CH_4 + O_2 \rightarrow C_3H_8 + 2H_2O$$

$$CH_4 + 2O_2 \rightarrow CO_2 + 2H_2O$$

As C_2H_6, C_3H_8 and CO_2 are the detected products for photocatalytic OCM, the oxygen balance can be calculated through the following equation:

$$Oxygen\ balance = \frac{n_{C2H6} \times 1/2\ +\ n_{C3H8}\ +\ n_{CO2} \times 2}{n_{O2}} \times 100\%$$

where n_{O2} is the molar number of consumed O_2, and n_{C2H6}, n_{C3H8} and n_{CO2} are the molar numbers of produced C_2H_6, C_3H_8 and CO_2, respectively. We specifically carried out the photocatalytic OCM test for 1 hour under the optimized reaction conditions. The amounts of the products were determined to be 0.052, 0.0025 and 0.0035 mmol for C_2H_4, C_3H_6 and CO_2, respectively. Meanwhile, the consumed O_2 amount was carefully determined by the peak area change of GC signals before and after reaction (see fig. S42 and S43), which was calculated through the equation of Mole of consumed oxygen = (GC signal area before reaction – GC signal area after reaction) / calibration coefficient × 10^{-2} × Gas flow rate × Time / 22.4 = 0.036 mmol. Therefore, the value of the oxygen balance is calculated to be 98%.

REFERENCES AND NOTES

1. J. Ma, C. Zhu, K. Mao, W. Jiang, J. Low, D. Duan, H. Ju, D. Liu, K. Wang, Y. Zang, S. Chen, H. Zhang, Z. Qi, R. Long, Z. Liu, L. Song, Y. Xiong, Sustainable methane utilization technology via photocatalytic halogenation with alkali halides. *Nat. Commun.* **14**, 1410 (2023).

2. D. Saha, H. A. Grappe, A. Chakraborty, G. Orkoulas, Postextraction separation, on-board storage, and catalytic conversion of methane in natural gas: A review. *Chem. Rev.* **116**, 11436–11499 (2016).

3. P. Tang, Q. Zhu, Z. Wu, D. Ma, Methane activation: The past and future. *Energ. Environ. Sci.* **7**, 2580–2591 (2014).

4. X. Li, C. Li, Y. Xu, Q. Liu, M. Bahri, L. Zhang, N. D. Browning, A. J. Cowan, J. Tang, Efficient hole abstraction for highly selective oxidative coupling of methane by Au-sputtered TiO_2 photocatalysts. *Nat. Energy* **8**, 1013–1022 (2023).

5. Z. Chen, S. Wu, J. Ma, S. Mine, T. Toyao, M. Matsuoka, L. Wang, J. Zhang, Non-oxidative coupling of methane: N-type doping of niobium single atoms in TiO_2-SiO_2 induces electron localization. *Angew. Chem. Int. Ed.* **60**, 11901–11909 (2021).

6. S. Wu, X. Tan, J. Lei, H. Chen, L. Wang, J. Zhang, Ga-doped and Pt-loaded porous TiO_2-SiO_2 for photocatalytic nonoxidative coupling of methane. *J. Am. Chem. Soc.* **141**, 6592–6600 (2019).

7. X. Yu, V. L. Zholobenko, S. Moldovan, D. Hu, D. Wu, V. V. Ordomsky, A. Y. Khodakov, Stoichiometric methane conversion to ethane using photochemical looping at ambient temperature. *Nat. Energy* **5**, 511–519 (2020).

8. C. Wang, X. Li, Y. Ren, H. Jiao, F. R. Wang, J. Tang, Synergy of Ag and AgBr in a pressurized flow reactor for selective photocatalytic oxidative coupling of methane. *ACS Catal.* **13**, 3768–3774 (2023).

9. S. Song, H. Song, L. Li, S. Wang, W. Chu, K. Peng, X. Meng, Q. Wang, B. Deng, Q. Liu, Z. Wang, Y. Weng, H. Hu, H. Lin, T. Kako, J. Ye, A selective Au-ZnO/TiO_2 hybrid photocatalyst for oxidative coupling of methane to ethane with dioxygen. *Nat. Catal.* **4**, 1032–1042 (2021).

10. X. Li, J. Xie, H. Rao, C. Wang, J. Tang, Platinum- and CuO_x-decorated TiO_2 photocatalyst for oxidative coupling of methane to C_2 hydrocarbons in a flow reactor. *Angew. Chem. Int. Ed.* **59**, 19702–19707 (2020).

11. Y. Wang, G. Hong, Y. Zhang, Y. Liu, W. Cen, L. Wang, Z. Wu, Photocatalytic oxidative coupling of methane over Au_1Ag single-atom alloy modified ZnO with oxygen and water vapor: Synergy of gold and silver. *Angew. Chem. Int. Ed.* **62**, e202310525 (2023).

12. X. Meng, X. Cui, N. P. Rajan, L. Yu, D. Deng, X. Bao, Direct methane conversion under mild condition by thermo-, electro-, or photocatalysis. *Chem* **5**, 2296–2325 (2019).

13. L. Li, Y. Y. Cai, G. D. Li, X. Y. Mu, K. X. Wang, J. S. Chen, Synergistic effect on the photoactivation of the methane C-H bond over Ga^{3+}-modified ETS-10. *Angew. Chem. Int. Ed.* **51**, 4702–4706 (2012).

14. G. Liu, Z. Zhu, S. M. Ciborowski, I. R. Ariyarathna, E. Miliordos, K. H. Bowen, Selective activation of the C-H Bond in methane by single platinum atomic anions. *Angew. Chem. Int. Ed.* **58**, 7773–7777 (2019).

15. W. Jiang, J. Low, K. Mao, D. Duan, S. Chen, W. Liu, C.-W. Pao, J. Ma, S. Sang, C. Shu, X. Zhan, Z. Qi, H. Zhang, Z. Liu, X. Wu, R. Long, L. Song, Y. Xiong, Pd-modified ZnO-Au enabling alkoxy intermediates formation and dehydrogenation for photocatalytic conversion of methane to ethylene. *J. Am. Chem. Soc.* **143**, 269–278 (2020).

16. J. Ma, Q. Zhang, Z. Chen, K. Kang, L. Pan, S. Wu, C. Chen, Z. Wu, J. Zhang, L. Wang, Design of frustrated Lewis pair in defective TiO_2 for photocatalytic non-oxidative methane coupling. *Chem Catal.* **2**, 1775–1792 (2022).

17. J. T. Grant, J. M. Venegas, W. P. McDermott, I. Hermans, Aerobic oxidations of light alkanes over solid metal oxide catalysts. *Chem. Rev.* **118**, 2769–2815 (2017).

18. N. Zhang, X. Feng, D. Rao, X. Deng, L. Cai, B. Qiu, R. Long, Y. Xiong, Y. Lu, Y. Chai, Lattice oxygen activation enabled by high-valence metal sites for enhanced water oxidation. *Nat. Commun.* **11**, 4066 (2020).

19. F. Zasada, J. Janas, W. Piskorz, M. Gorczyńska, Z. Sojka, Total oxidation of lean methane over cobalt spinel nanocubes controlled by the self-adjusted redox state of the catalyst: Experimental and theoretical account for Interplay between the Langmuir–Hinshelwood and Mars–Van Krevelen mechanisms. *ACS Catal.* **7**, 2853–2867 (2017).

20. Z. Liu, E. Huang, I. Orozco, W. Liao, R. M. Palomino, N. Rui, T. Duchoň, S. Nemšák, D. C. Grinter, M. Mahapatra, P. Liu, J. A. Rodriguez, S. D. Senanayake, Water-promoted interfacial pathways in methane oxidation to methanol on a CeO_2-Cu_2O catalyst. *Science* **368**, 513–517 (2020).

21. P. Schwach, X. Pan, X. Bao, Direct conversion of methane to value-added chemicals over heterogeneous catalysts: Challenges and prospects. *Chem. Rev.* **117**, 8497–8520 (2017).

22. P. Wang, R. Shi, Y. Zhao, Z. Li, J. Zhao, J. Zhao, G. I. N. Waterhouse, L. Z. Wu, T. Zhang, Selective photocatalytic oxidative coupling of methane via regulating methyl intermediates over Metal/ZnO nanoparticles. *Angew. Chem. Int. Ed.* **62**, e202304301 (2023).

23. X. Li, C. Wang, J. Yang, Y. Xu, Y. Yang, J. Yu, J. J. Delgado, N. Martsinovich, X. Sun, X.-S. Zheng, W. Huang, J. Tang, PdCu nanoalloy decorated photocatalysts for efficient and selective oxidative coupling of methane in flow reactors. *Nat. Commun.* **14**, 6343 (2023).

24. C. Liu, B. Qian, T. Xiao, C. Lv, J. Luo, J. Bao, Y. Pan, Illustrating the fate of methyl radical in photocatalytic methane oxidation over Ag-ZnO by in situ synchrotron radiation photoionization mass spectrometry. *Angew. Chem. Int. Ed.* **62**, e202304352 (2023).

25. Y. Wang, Y. Zhang, Y. Liu, Z. Wu, Photocatalytic oxidative coupling of methane to ethane using water and oxygen on Ag_3PO_4-ZnO. *Environ. Sci. Technol.* **57**, 11531–11540 (2023).

26. J. Xie, Y. Jiang, S. Li, P. Xu, Q. Zheng, X. Fan, H. Peng, Z. Tang, Stable photocatalytic coupling of methane to ethane with water vapor using TiO_2 supported ultralow loading AuPd nanoparticles. *Acta Phys.-Chim. Sin.* **39**, 2306037 (2023).

27. G. Zhai, S. Liu, S. Si, Y. Liu, H. Zhang, Y. Mao, M. Zhang, Z. Wang, H. Cheng, P. Wang, Z. Zheng, Y. Dai, B. Huang, oxygen vacancies enhanced ozonation toward phenol derivatives removal over O_v-Bi_2O_3. *ACS ES&T Water* **2**, 1725–1733 (2022).

28. G. Zhai, Y. Liu, L. Lei, J. Wang, Z. Wang, Z. Zheng, P. Wang, H. Cheng, Y. Dai, B. Huang, Light-promoted CO_2 conversion from epoxides to cyclic carbonates at ambient conditions over a bi-based metal–organic framework. *ACS Catal.* **11**, 1988–1994 (2021).

29. S. Si, H. Shou, Y. Mao, X. Bao, G. Zhai, K. Song, Z. Wang, P. Wang, Y. Liu, Z. Zheng, Y. Dai, L. Song, B. Huang, H. Cheng, Low-coordination single Au atoms on ultrathin $ZnIn_2S_4$ nanosheets for selective photocatalytic CO_2 reduction towards CH_4. *Angew. Chem. Int. Ed.* **61**, e202209446 (2022).

30. S. Zou, B. Lou, K. Yang, W. Yuan, C. Zhu, Y. Zhu, Y. Du, L. Lu, J. Liu, W. Huang, B. Yang, Z. Gong, Y. Cui, Y. Wang, L. Ma, J. Ma, Z. Jiang, L. Xiao, J. Fan, Grafting nanometer metal/oxide interface towards enhanced low-temperature acetylene semi-hydrogenation. *Nat. Commun.* **12**, 5770 (2021).

31. B. Hammer, J. K. Nørskov, Electronic factors determining the reactivity of metal surfaces. *Surf. Sci.* **343**, 211–220 (1995).

32. B. Hammer, J. K. Norskov, Why gold is the noblest of all the metals. *Nature* **376**, 238–240 (1995).

33. T. Ban, X.-Y. Yu, H.-Z. Kang, Z.-Q. Huang, J. Li, C.-R. Chang, Design of SA-FLP dual active sites for nonoxidative coupling of methane. *ACS Catal.* **13**, 1299–1309 (2023).

34. C. Li, Q. Xin, FT-IR spectroscopic investigation of methane adsorption on cerium oxide. *J. Phys. Chem. C* **96**, 7714–7718 (1992).

35. F. F. Tao, J.-J. Shan, L. Nguyen, Z. Wang, S. Zhang, L. Zhang, Z. Wu, W. Huang, S. Zeng, P. Hu, Understanding complete oxidation of methane on spinel oxides at a molecular level. *Nat. Commun.* **6**, 7798 (2015).

36. X. Yu, V. De Waele, A. Löfberg, V. Ordomsky, A. Y. Khodakov, Selective photocatalytic conversion of methane into carbon monoxide over zinc-heteropolyacid-titania nanocomposites. *Nat. Commun.* **10**, 700 (2019).

37. W. Zhang, C. Fu, J. Low, D. Duan, J. Ma, W. Jiang, Y. Chen, H. Liu, Z. Qi, R. Long, Y. Yao, X. Li, H. Zhang, Z. Liu, J. Yang, Z. Zou, Y. Xiong, High-performance photocatalytic nonoxidative conversion of methane to ethane and hydrogen by heteroatoms-engineered TiO_2. *Nat. Commun.* **13**, 2806 (2022).

38. K. Mudiyanselage, S. D. Senanayake, L. Feria, S. Kundu, A. E. Baber, J. Graciani, A. B. Vidal, S. Agnoli, J. Evans, R. Chang, S. Axnanda, Z. Liu, J. F. Sanz, P. Liu, J. A. Rodriguez, D. J. Stacchiola, Importance of the metal-oxide interface in catalysis: In situ studies of the water-gas shift reaction by ambient-pressure x-ray photoelectron spectroscopy. *Angew. Chem. Int. Ed.* **52**, 5101–5105 (2013).

39. A. Yee, S. J. Morrison, H. Idriss, A study of the reactions of ethanol on CeO_2 and Pd/CeO_2 by steady state reactions, temperature programmed desorption, and in situ FT-IR. *J. Catal.* **186**, 279–295 (1999).

40. Z. Jiao, M. Shang, J. Liu, G. Lu, X. Wang, Y. Bi, The charge transfer mechanism of Bi modified TiO_2 nanotube arrays: TiO_2 serving as a "charge-transfer-bridge". *Nano Energy* **31**, 96–104 (2017).

41. A. Naldoni, M. Altomare, G. Zoppellaro, N. Liu, Š. Kment, R. Zbořil, P. Schmuki, Photocatalysis with reduced TiO_2: From black TiO_2 to cocatalyst-free hydrogen production. *ACS Catal.* **9**, 345–364 (2018).

42. E. Carter, A. F. Carley, D. M. Murphy, Evidence for O_2^- radical stabilization at surface oxygen vacancies on polycrystalline TiO_2. *J. Phys. Chem. C* **111**, 10630–10638 (2007).

43. G. Kresse, J. Furthmüller, Efficient iterative schemes for ab initio total-energy calculations using a plane-wave basis set. *Phys. Rev. B* **54**, 11169–11186 (1996).

44. G. Kresse, D. Joubert, From ultrasoft pseudopotentials to the projector augmented-wave method. *Phys. Rev. B* **59**, 1758–1775 (1999).

45. J. P. Perdew, K. Burke, M. Ernzerhof, Generalized gradient approximation made simple. *Phys. Rev. Lett.* **77**, 3865–3868 (1996).

46. M. Dion, H. Rydberg, E. Schröder, D. C. Langreth, B. I. Lundqvist, Van der Waals density functional for general geometries. *Phys. Rev. Lett.* **92**, 246401 (2004).

47. K. Lee, É. D. Murray, L. Kong, B. I. Lundqvist, D. C. Langreth, Higher-accuracy van der Waals density functional. *Phys. Rev. B* **82**, 081101 (2010).

48. J. K. Nørskov, J. Rossmeisl, A. Logadottir, L. Lindqvist, J. R. Kitchin, T. Bligaard, H. Jónsson, Origin of the overpotential for oxygen reduction at a fuel-cell cathode. *J. Phys. Chem. B* **108**, 17886–17892 (2004).

49. Y. Chen, Y. Zhao, D. Liu, G. Wang, W. Jiang, S. Liu, W. Zhang, Y. Li, Z. Ma, T. Shao, H. Liu, X. Li, Z. Tang, C. Gao, Y. Xiong, Continuous flow system for highly efficient and durable photocatalytic oxidative coupling of methane. *J. Am. Chem. Soc.* **146**, 2465–2473 (2024).
